# Identification of Dietary Bioflavonoids as Potential Inhibitors against KRAS G12D Mutant—Novel Insights from Computer-Aided Drug Discovery

**DOI:** 10.3390/cimb45030137

**Published:** 2023-03-06

**Authors:** Prasanna Srinivasan Ramalingam, Purushothaman Balakrishnan, Senthilnathan Rajendran, Arunachalam Jothi, Rajasekaran Ramalingam, Sivakumar Arumugam

**Affiliations:** 1 Protein Engineering Lab, School of Biosciences and Technology, VIT University, Vellore 632014, Tamil Nadu, India; 2 TanBio R and D Solution, Thanjavur 613403, Tamil Nadu, India; 3 Department of Bioinformatics, School of Chemical and Biotechnology, SASTRA Deemed University, Thanjavur 613401, Tamil Nadu, India; 4 Quantitative Biology Lab, School of Biosciences and Technology, VIT University, Vellore 632014, Tamil Nadu, India

**Keywords:** cancer, KRAS G12D, inhibitor, flavonoids, ADMET, mutant inhibitor

## Abstract

The KRAS G12D mutation is very frequent in many cancers, such as pancreatic, colon and lung, and has remained undruggable for the past three decades, due to its smooth surface and lack of suitable pockets. Recent small pieces of evidence suggest that targeting the switch I/II of KRAS G12D mutant could be an efficient strategy. Therefore, in the present study, we targeted the switch I (residues 25–40) and switch II (residues 57–76) regions of KRAS G12D with dietary bioflavonoids in comparison with the reference KRAS SI/II inhibitor BI-2852. Initially, we screened 925 bioflavonoids based on drug-likeness properties, and ADME properties and selected 514 bioflavonoids for further studies. Molecular docking resulted in four lead bioflavonoids, namely 5-Dehydroxyparatocarpin K (L1), Carpachromene (L2), Sanggenone H (L3), and Kuwanol C (L4) with binding affinities of 8.8 Kcal/mol, 8.64 Kcal/mol, 8.62 Kcal/mol, and 8.58 Kcal/mol, respectively, in comparison with BI-2852 (−8.59 Kcal/mol). Further steered-molecular dynamics, molecular-dynamics simulation, toxicity, and in silico cancer-cell-line cytotoxicity predictions significantly support these four lead bioflavonoids as potential inhibitors of KRAS G12D SI/SII inhibitors. We finally conclude that these four bioflavonoids have potential inhibitory activity against the KRAS G12D mutant, and are further to be studied in vitro and in vivo, to evaluate their therapeutic potential and the utility of these compounds against KRAS G12D mutated cancers.

## 1. Introduction

RAS (RAt sarcoma viral oncogene homolog) proteins are small guanine nucleotide triphosphatases (GTPases) that act as a molecular switch in signal transduction by toggling between guanosine triphosphate (GTP)-bound active state and guanosine diphosphate (GDP)-bound inactive state [1,2]. The GTP to GDP (GTP hydrolysis) conversion is mediated by GTPase-activating proteins (GAPs), and the GDP–GTP exchange is facilitated by guanine nucleotide exchange factors (GEFs) such as Son of Sevenless (SOS) [3,4]. Ras proteins play a major role in cell growth, proliferation, differentiation, and survival, by their involvement in various signaling pathways such as phosphoinositide-3-kinase (PI3K), diacylglycerol (DAG), phospholipase C (PLC), c-Jun amino-terminal kinase (JNK), extracellular signal-regulated kinase (ERK), mitogen-activated ERK kinase (MEK), and, most importantly, by mitogen-activated protein kinases (MAPK) (also called MAPK/ERK or Ras-Raf-MEK-ERK pathway) signaling pathways [5]. RAS mutations are very common, and account for nearly 30% of all cancer types, especially those involved in the aggressive metastatic malignancies of pancreatic (95%), lung (35%), and colorectal (45%) cancers [6].

Generally, RAS proteins have three isoforms, namely Kirsten Rat sarcoma viral oncogene homolog (KRAS), Harvey Rat sarcoma viral oncogene homolog (HRAS), and Neuroblastoma Rat sarcoma viral oncogene homolog (NRAS) [7,8,9]. The KRAS, NRAS and HRAS isoforms have mutation frequencies of 85%, 11%, and 4%, respectively, and the G12, G13, and Q61 are the mutational hotspots in all three isoforms [10,11]. The mutations at these residues remain in the GTP-bound active state, and rapidly trigger the proliferation and survival of cancer cells.

**Figure 1:**
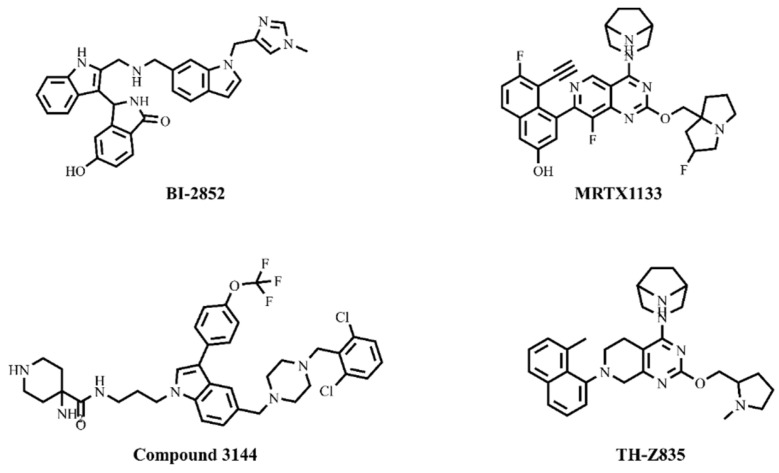
2D structures of KRAS G12D inhibitors.

Among all the isoforms, KRAS have high mutation frequencies, with mutational frequencies at G12, G13, and Q61 of 83%, 14%, and 2%, respectively [12]. Notably, the G12 position (83%) of the KRAS has high mutational frequency with a single nucleotide substitution—the missense mutation (97.5% are point mutation) such as G12D (33%), G12V (23%), G12C (12%), G12A (5%), G12S (4%), and G12R (2%) respectively [13]. Comparatively, the KRAS G12D (the single-nucleotide change from glycine (G) to aspartic acid (D) at 12th position (c.35G>A)) mutant has higher mutational frequencies than the other forms of KRAS [12,13,14] (https://cancer.sanger.ac.uk/cmc/home (accessed on 20 September 2022)). Of note, the KRAS G12D mutant promotes proliferative signaling, cell-replicative immortality, tumor-promoting inflammation, angiogenesis, a change in cellular energetics, invasion and metastasis, and suppresses apoptosis by down-regulating the thymine-DNA glycosylase (TDG) [14]. For the past 40 years, the KRAS mutants have seemed undruggable, due to their smooth surface and lack of suitable pocket for the inhibitor. Several studies have been carried out to target KRAS, which resulted in the successful development of KRAS G12C inhibitor Sotorasib (AMG 510), with FDA approval, in 2021 [15]. AMG 510 forms an irreversible covalent bond with the Cys12 residue in the H95/Y96/Q99 cryptic pocket, which includes the switch II of KRAS mutants [15]. Simultaneously, direct KRAS G12D mutant-targeting inhibitors such as MRTX1133, Compound 3144, and a cyclic peptide–KD2 have been developed and studied, which still lack effective inhibition [16]. Although several studies have been carried out, the KRAS G12D has a very high mutation-frequency and challenge it as undruggable, due to the absence of reactive cysteine at the 12th position (as KRAS G12C mutant). Interestingly, some potential inhibitors such as MRTX1133, Compound 3144, TH-Z835, and BI-2852 have been reported to target the switch I and switch II of the KRAS mutants, which seems a promising strategy to inhibit the KRAS G12D mutant [16,17,18,19]. The 2D structures of MRTX1133, Compound 3144, TH-Z835, and BI-2852 are shown in Figure 1.

**Figure 2:**
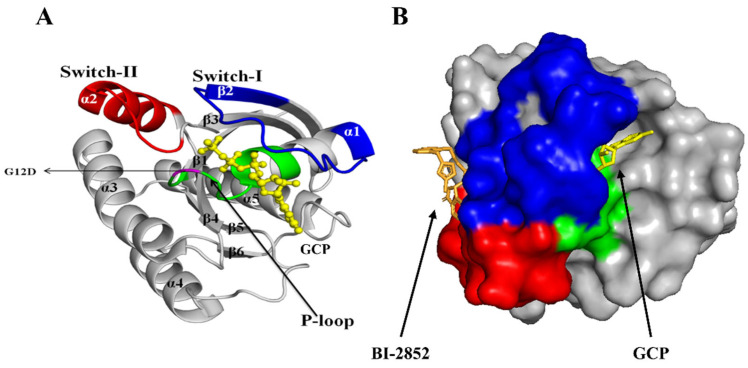
KRAS G12D mutant protein architecture: (**A**) A 3-D cartoon view of the GTP-bound KRAS G12D mutant protein (PBD ID: 6GJ8) was visualized, and its α-helices (α1, α2, α3, α4, α5), β-sheets (β1, β2, β3, β4, β5, β6), switches (SI and SII) and P-loop are represented. The missense substitution mutation is present in the 12th position (in the P-loop) where the glycine (G) is substituted by aspartic acid (D). (**B**) Co-crystallized structure of phosphomethylphosphonic acid guanylate ester (GCP) (yellow) and BI-2852 (bright orange) in KRAS G12D mutant protein (PBD ID: 6GJ8). The P-loop (residues 10–17), switch I (residues 25–40), and switch II (residues 57–76) are shown in green, blue, and red, respectively. The SI/SII binding site of BI-2852 was selected for the molecular-docking studies.

BI-2852 was discovered as a KRAS switch I/II inhibitor, which inhibits the GTP bound-KRAS G12D at 740 nM (nearly a 10-fold increase compared with the KRAS WT). It was reported to inhibit the KRAS: SOS interaction, and reduced the phosphorylation rates of its downstream effectors [20]. The three-dimensional structure of the KRAS G12D mutant protein consists of a 172 amino-acid length with structural features such as α-helices (α1, α2, α3, α4, α5), β-sheets (β1, β2, β3, β4, β5, β6), switches (SI and SII) and P-loop (where G12D mutant is present), as shown in Figure 2. The regions including switch I (residues 25–40) and switch II (residues 57–76) were considered as the pocket for the screening of suitable inhibitors in comparison with BI-2852 as the reference KRAS SI/II inhibitor [21].In the end, flavonoids are polyphenols that are highly reported for their anticancer potential against various cancers, such as lung, breast, colon, prostate, and ovarian [22]. Flavonoids are widely reported to have strong anticancer properties by possessing apoptosis and autophagy induction, cell-cycle arrest at G1/S and G2/M phases, mediating cellular signaling, including MAPK & PI3-K pathways, intracellular-ROS-modulatory potential, an anti-invasiveness nature and antiproliferation properties [23,24]. All the reported KRAS G12D inhibitors were identified through a fragment-based approach, and still, to the best of our knowledge, there were no studies on the evaluation of the inhibitory potential of flavonoids against KRAS G12D. Therefore, in the present study, we explore the therapeutic potential of bioflavonoids against the KRAS G12D mutant by examining their drug-likeness properties, ADME properties, binding affinities, steered molecular dynamics, molecular-dynamics simulation, possible activity against kinases, toxicity, and in silico cancer-cell-line cytotoxicity profiles.

## 2. Methods

### 2.1. Protein and Ligand Preparation

**Figure 3:**
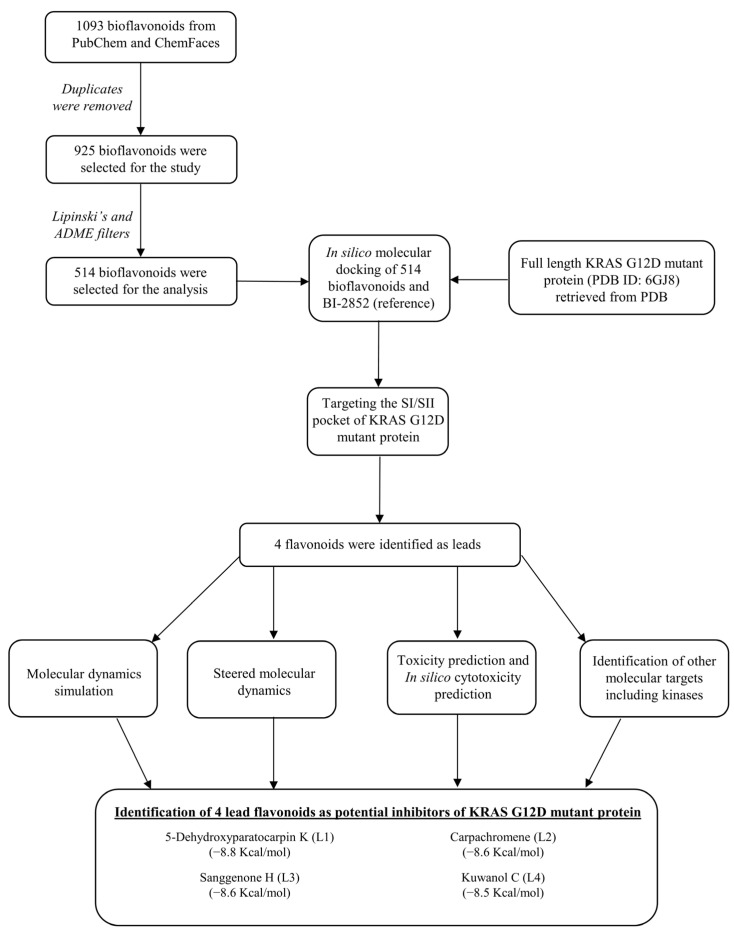
Schematic representation of the workflow.

The three-dimensional crystal structure of the full length KRAS G12D mutant protein (PDB ID: 6GJ8) co-crystallized with GTP and BI-2852 was retrieved from the PDB data bank [25] (https://www.rcsb.org/ (accessed on 17 December 2022)). The excess water molecules and heteroatoms were removed from the 172-amino-acid-length KRAS G12D mutant protein. The three-dimensional crystal structure of BI-2852 (KRAS switch I/II pocket inhibitor), and a total of 1093 flavonoids that have been experimentally proven for their therapeutic potential were retrieved from PubChem database [26] (https://pubchem.ncbi.nlm.nih.gov/ (accessed on 25 September 2022)) and ChemFaces (http://www.chemfaces.com/ (accessed on 25 September 2022)). The duplicates were then removed, and finally 925 flavonoids were taken for the study. The SDF-file format of all the 925 flavonoids were converted into PDB-file format, using open babel web server [27] (http://www.cheminfo.org/Chemistry/Cheminformatics/FormatConverter/index.html (accessed on 26 September 2022)). Then the energies of the ligands were minimized in the Avogadro program version 1.2.0, using Universal Force Field (UFF) by the steepest-descent method [28] (https://avogadro.cc/ (accessed on 28 September 2022)), and the ligands were prepared for molecular docking. The complete workflow of the study is provided in Figure 3.

### 2.2. Drug-Likeness and ADME Properties

The drug-likeness and ADME properties of all the 925 flavonoids were evaluated using the SwissADME web tool [29] (http://www.swissadme.ch/ (accessed on 1 October 2022)). Lipinski’s rule, the rule of five (RO5), is widely used to predict the small molecules which are biologically active and orally bioavailable [30]. The RO5 states that the drug-likeness properties are that the molecular mass should be less than 500 Dalton, the hydrogen-bond donors should be fewer than 5, hydrogen-bond acceptors should be fewer than 10, and the partition coefficient log P or lipophilicity should be less than 5 [31]. In addition, the absorption, distribution, metabolism, and excretion (ADME) properties determine the pharmacokinetic parameters of the drugs by addressing their safety and efficacy [32]. The drug-likeness and ADME-properties evaluation resulted in 514 bioflavonoids, which were further taken for the molecular-docking studies.

### 2.3. Molecular Docking

To evaluate the binding affinities of the selected flavonoids towards the KRAS G12D mutant protein, molecular docking was performed using AutoDock Vina [33] (http://vina.scripps.edu/ (accessed on 19 December 2022)). The grid points were set as 60 Å × 60 Å × 60 Å in X, Y, and Z dimensions, and the center grid box was set as 12.82 Å, 16.258 Å, and −15.154 Å in X, Y, and Z directions, with a point spacing of 0.375 Å; all other parameters were set to default. The same coordinates in the BI-2852 binding position in the KRAS G12D (PDB ID: 6GJ8) which targets switch I (residues 25–40), and switch II (residues 57–76), as shown in Figure 2. Then the selected 514 bioflavonoids and the BI-2852 were docked at the above-mentioned pocket, to predict their binding affinities. The protein-ligand complexes were visualized using Pymol and their interactions were predicted using Ligplot.

### 2.4. Steered Molecular Dynamics

Steered molecular dynamics relies on the ideology of measuring the pulling energy spent on the system to pull out the ligand from the protein—ligand complex as a measure of binding affinity. In simple words, the larger the energy spent to pull out the ligand, the higher the protein—ligand affinity, and the less the energy spent to pull out the ligand, the less the protein—ligand affinity. The steered molecular dynamics (SMD) of the top 4 lead flavonoids filtered from the above studies, along with the BI-2852 (KRAS switch I/II pocket inhibitor) was performed using YASARA software [34] (http://www.yasara.org/md.htm (accessed on 23 December 2022)). At 298K and 7 pH, the amber force field with a water-filled cubic box with 0.997 g/mL solvent density, 0.9% NaCl, and 75 Å × 75 Å × 75 Å as x, y, and z dimensions was constructed [35]. The protein’s center of mass was kept constant, and the ligands were pulled with a constant pulling acceleration of 2000 pm/ps^2^ up to 15 Å distance, and the time taken was calculated for every 1 ps. This SMD evaluation reveals how efficient the binding patterns of these flavonoids with KRAS G12D mutant are in comparison with the BI-2852. 

### 2.5. Molecular-Dynamics Simulation

The molecular-dynamics simulation (MDS) of protein-ligand complexes is generally employed to predict the stability and mode of binding of the ligand to the protein in its dynamic state [36]. Here, the molecular-dynamics simulation of the BI-2852 and the 4 lead flavonoids bound at the SI/II region of the KRAS G12D mutant was performed using the YASARA Structure package using macro MD_Analyze.mcr. The periodic cubic cell for the simulation was constructed using an AMBER force field with extended boundaries up to 8 Å away from the complex, filled with water (water bumps = 1 Å); 0.9% NaCl was added to maintain the physiological condition, and the density of 0.997 g mL^−1^, and the H-bond network was also optimized [37,38]. The conformational stress of the entire system was reduced by minimizing the energy by the steepest-descent method up to a convergence of <0.01 kcal/mol Å. The MDS was performed using an NPT ensemble (constant temperature and constant pressure) with 298 K temperature and 1 atm pressure. The root-mean-square deviation (RMSD), root-mean-square fluctuation (RMSF), radius of gyration (Rg), and the solvent-accessible surface area (SASA) of the docked complexes of BI-2852 and 4 lead flavonoids with the KRAS G12D mutant and KRAS G12D mutant alone (apoprotein) was predicted from the molecular-dynamics simulation studies, which reveal the ligand—protein stability over the period of time.

### 2.6. SwissTargetPrediction Analysis

The probable protein targets of the top 4 lead flavonoids were predicted using the SwissTargetPrediction web server [39] (http://www.swisstargetprediction.ch/ (accessed on 26 December 2022)). The canonical smiles of the 4 lead flavonoids were entered, and the filter of species restricted to *Homo sapiens* alone, which provided various molecular targets such as cytosolic proteins, kinases, oxidoreductases, cytochrome p450 enzymes, GPCRs, nuclear receptors, ion channels, and membrane proteins. Of note, the SwissTargetPrediction webserver has 376 k compounds interactions with various proteins with experimental data reported in *Homo sapiens*, *Mus musculus*, and *Rattus norvegicus*.

### 2.7. In Silico Cytotoxicity Prediction

The in silico cytotoxicity prediction of the 4 lead flavonoids against various tumor cell lines was predicted using the CLC-Pred web server [40] (http://way2drug.com/Cell-line/ (accessed on 26 December 2022)). CLC-Pred uses the PASS (prediction-of-activity-spectra-for-substances) algorithm, to predict the cytotoxic profiles of chemicals against various cancer cell lines using the structure–activity relationship (SAR). The canonical smiles of the 4 lead flavonoids and BI-2852 were entered, and their Pa (probability of activeness) and the Pi (probability of inactiveness) to be cytotoxic against the cancer cells were calculated. Of note, it contained nearly 59k chemicals with SAR and experimental data against 278 different cancer cell lines, with 93% accuracy. In addition, the toxicity parameters such as human hepatotoxicity (H-HT), drug-induced liver injury (DILI), AMES toxicity, rat oral acute toxicity (ROAT), FDA maximum (recommended) daily dose (FDAMDD), and activities against androgen receptor (NR-AR), estrogen receptor (NR-ER), peroxisome proliferator-activated receptor gamma (NR-PPAR-γ), antioxidant-response element (SR-ARE), carcinogenicity, golden triangle and toxicophores of the top 4 leads and BI-2852 were evaluated, using ADMETLAB 2.0 [41] (https://admetmesh.scbdd.com/service/evaluation/index (accessed on 26 December 2022)).

## 3. Results

### 3.1. Evaluation of Lipinski’s Rule and ADME Properties

The drug-likeness and ADME properties of the compounds govern their safety and efficacy in the drug-discovery processes. The SwissADME web server was utilized for the prediction of drug-likeness and absorption, distribution, metabolism, and excretion (ADME) properties of all the 925 flavonoids. The physicochemical properties such as molecular weight, aromatic heavy atoms, H-bond donor, H-bond acceptor, rotatable bonds, molar refractivity, and topological polar surface area were evaluated. In addition, the ADME properties such as lipophilicity, hydrophilicity, pharmacokinetics, gastrointestinal absorption, and bioavailability of all the 925 bioflavonoids were evaluated. The synthetic accessibility on a scale of 10, indicating the laboratory-synthesis possibilities of the compounds, was also evaluated. This evaluation resulted in the 514 flavonoids passing Lipinski’s rule and satisfying the drug-likeness properties, and passing all the ADME parameters. The drug-likeness properties and the ADME of the top 4 lead bioflavonoids resulting from the molecular-docking studies are shown in Table 1 and Table 2, respectively. The synthetic accessibility of all 4 lead flavonoids lies in the 3–5 range, and indicates that these can also be chemically synthesized, and can be structurally modified for the enhancement of binding affinities towards KRAS G12D, using medicinal-chemistry approaches.

### 3.2. Molecular-Docking Analysis

**Figure 4:**
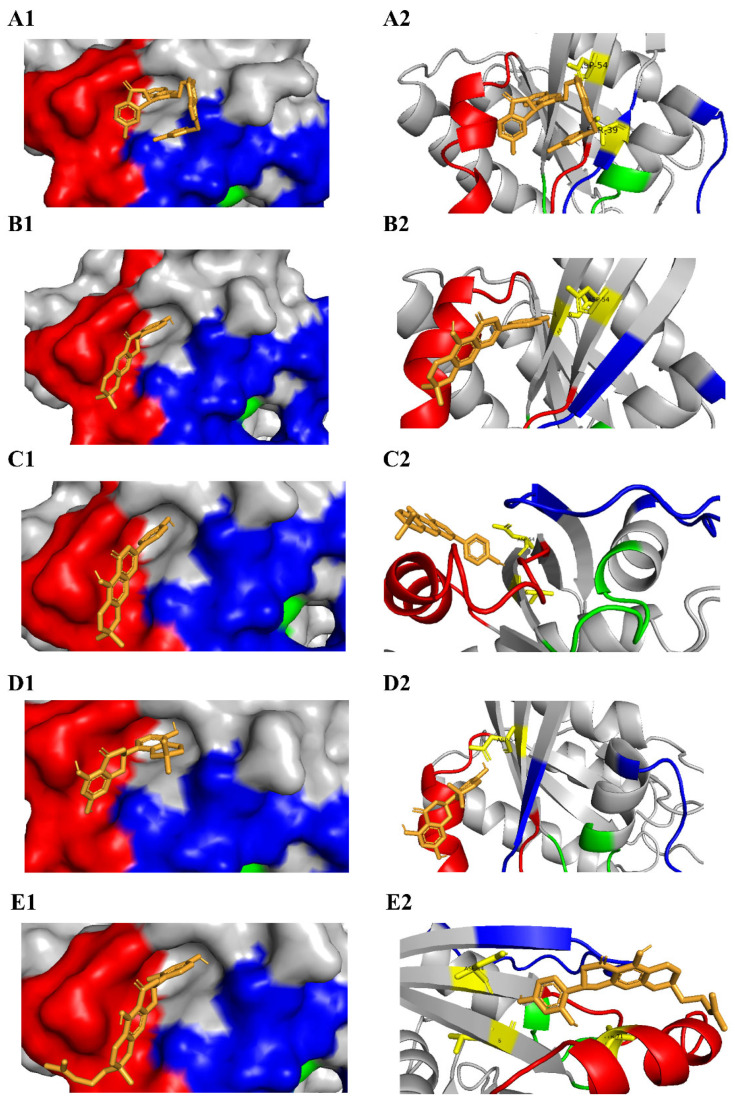
Docked poses of 4 leads with KRAS G12D mutant protein: The P-loop (residues 10–17), switch I (residues 25–40), and switch II (residues 57–76) are shown in green, blue and red, respectively. In the left panel, the docked positions of BI-2852 (reference), 5-Dehydroxyparatocarpin K (L1), Carpachromene (L2), Sanggenone H (L3), and Kuwanol C (L4) are represented as (**A1**–**E1**), respectively. In the right panel, the docked pose, along with their H-bond residues (yellow) in the protein of BI-2852, L1, L2, L3, and L4, are represented as (**A2**–**E2**), respectively.

The binding affinities of the selected 514 flavonoids against the KRAS G12D mutant protein were determined by in silico molecular docking using AutoDock Vina. The binding energies of all the 514 flavonoids, along with their canonical smiles, are provided in Supplementary Table S1. All the flavonoids mostly showed good binding affinities, with binding energies ranging from −8.8 Kcal/mol to −5 Kcal/mol. Although all the flavonoids showed good affinity against the KRAS G12D mutant, it is very important to show significant affinity towards the KRAS SI/SII pocket such as BI-2852, to yield the inhibition activity. These 514 bioflavonoids, along with the BI-2852, were docked at the same co-ordinates of the crystallized structure of BI-2852 with KRAS G12D (PBD ID: 6GJ8). The docked poses and the molecular interactions of BI-2852 in crystallized form and the docked form were superimposed and possess similar type of interactions (the indole group near to the isatin group has a closer interaction in the SI/II region) as shown in Supplementary Figure S1. The molecular-docking results resulted in the identification of 4 potential lead compounds, namely 5-Dehydroxyparatocarpin K (L1), Carpachromene (L2), Sanggenone H (L3), and Kuwanol C (L4), which showed binding affinities of −8.8 Kcal/mol, −8.64 Kcal/mol, −8.62 Kcal/mol, and −8.58 Kcal/mol, respectively, against the KRAS G12D mutant, in comparison with BI-2852 (−8.59 Kcal/mol). The binding energies (Kcal/mol), inhibitory constant Ki (µM), and the interacting amino-acid residues of these 4 lead flavonoids and BI-2852 around the KRAS G12D switch I/II pocket were evaluated, and tabulated in Table 3. The binding poses of these compounds were also visualized, and are shown in Figure 4. The left panel shows the compounds’ binding poses at the switch I/II pocket in surface view, and the right panel shows their interaction in cartoon view, along with the H-bond forming residues (shown in yellow) in the KRAS G12D mutant protein, as shown in Figure 4.

### 3.3. Binding Interactions of Lead Flavonoids

**Figure 5:**
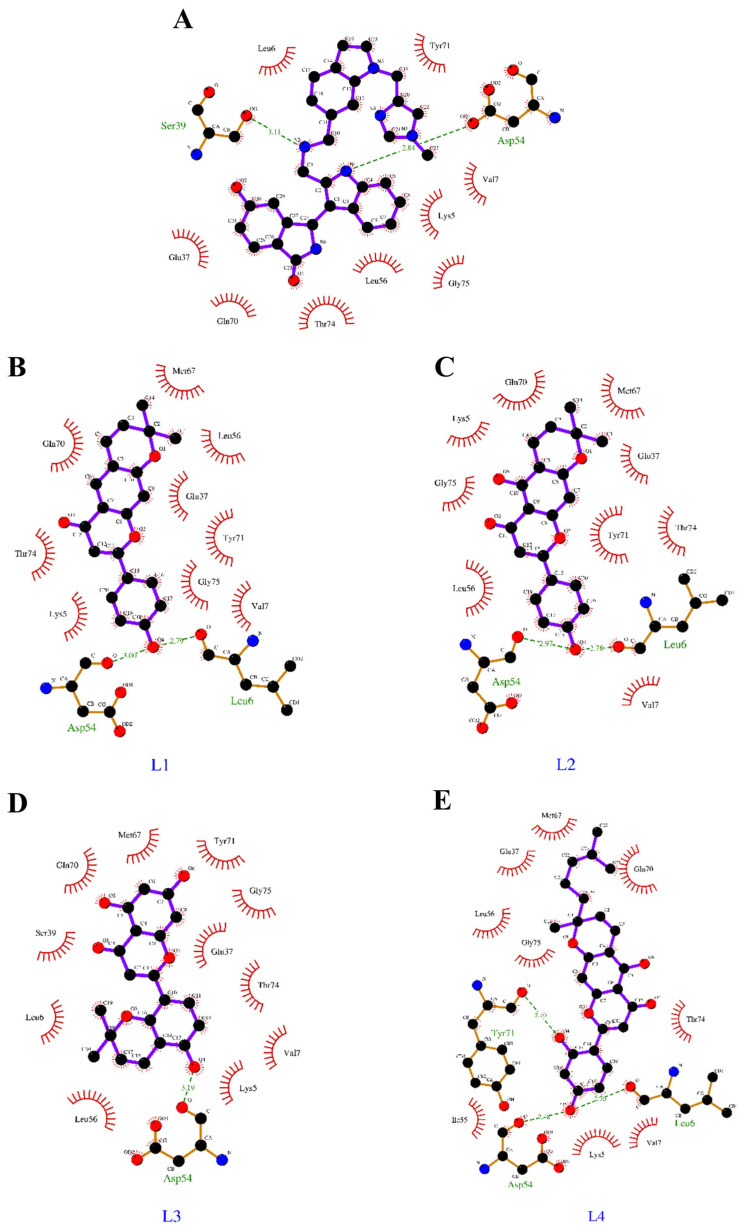
Ligplot interactions of 4 leads with KRAS G12D mutant protein: The protein–ligand interactions of BI-2852 (reference), 5-Dehydroxyparatocarpin K (L1), Carpachromene (L2), Sanggenone H (L3), and Kuwanol C (L4) are represented as (**A**–**E**), respectively. The H-bonds and other hydrophobic and electrostatic interactions of these compounds are also shown.

The intramolecular H-bonds, intramolecular electrostatic and hydrophobic interactions of 5-Dehydroxyparatocarpin K (L1), Carpachromene (L2), Sanggenone H (L3), and Kuwanol C (L4) along with the BI-2852 (KRAS switch I/II pocket inhibitor) against the KRAS G12D mutant were evaluated. The intermolecular-hydrogen-bond interactions of four leads, along with the BI-2852, are shown in Table 4, and the intramolecular-electrostatic and hydrophobic interactions of these compounds are shown in Table 5. Our results revealed that BI-2852 (reference) showed two H-bonds with Ser39 and Asp54, one electrostatic interaction with Asp54, and one hydrophobic interaction with Leu56; L1 showed two H-bonds with Leu6 and Asp54, and three hydrophobic interactions with Lys5, Leu56, and Met67; L2 showed two H-bonds with Leu6 and Asp54, and three hydrophobic interactions with Lys5, Leu56, Met67; L3 showed one H-bond with Asp54, and one electrostatic, and three hydrophobic interactions with Lys5, Leu56 and Met67; L4 showed three H-bonds in total with Leu6, Asp54 and Tyr71, and three hydrophobic interactions in total with Lys5, Leu56, and Met67 respectively, as shown in Table 4 and Table 5. All four leads and BI-2852 showed an H-bond interaction with Asp54 in common, and hydrophobic interactions with Leu56. Leu56 and Met67 residues are responsible for the alkyl hydrophobic interaction of the four 4 leads, and Lys5 and Leu56 residues are responsible for the mixed Pi-alkyl hydrophobic interaction of the four leads, while Leu56 is the only residue with a mixed Pi-alkyl hydrophobic interaction in BI-2852, and no key residues were observed for the alkyl hydrophobic interaction of BI-2852. Overall, all four lead flavonoids showed excellent binding affinities towards the KRAS G12D mutant protein, by interacting with the switch I and switch II pocket. The binding interactions of the compounds were evaluated using Ligplot, and are shown in Figure 5.

### 3.4. Steered Molecular Dynamics

**Figure 6:**
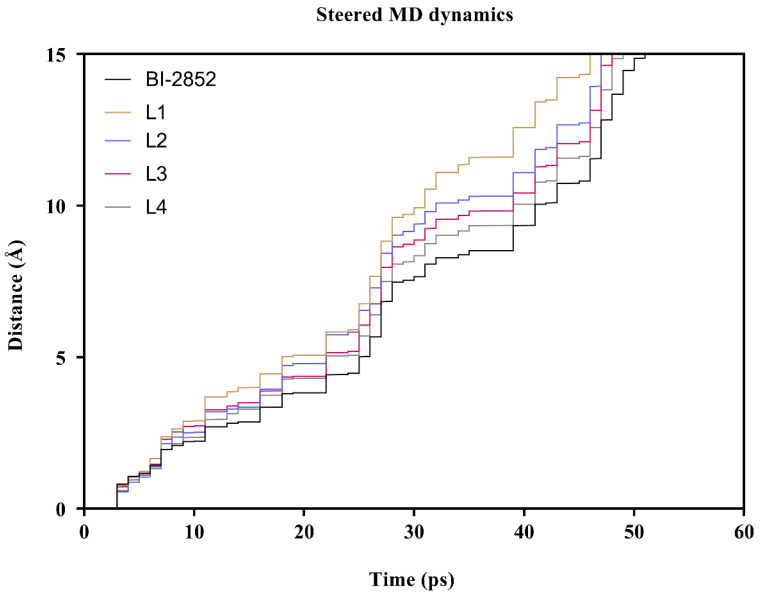
Dissociation of BI-2852 and lead flavonoids from the KRAS G12D mutant protein: the time taken (in picoseconds) to pull out the ligands, BI-2852 (reference compound), 5-Dehydroxyparatocarpin K (L1), Carpachromene (L2), Sanggenone H (L3), and Kuwanol C (L4) from the KRAS G12D mutant protein’s center to 15 Å apart was calculated and their dissociation trajectories were represented.

The steered molecular dynamics (SMD) of the top four lead flavonoids and BI-2852 were performed, using YASARA software. The cubic box was constructed with an amber force field and with the mentioned parameters, and the time taken to dissociate the ligand molecule to 15 Å distance apart from the protein’s center mass was evaluated as the measure of the binding affinity of the ligands toward the KRAS G12D mutant protein. The time taken to dissociate the top four p4 lead flavonoids and BI-2852 was calculated, and is given in Table 6, and the trajectory analysis at every 1 ps is shown in Figure 6. At 2000 pm/ps^2^ pulling acceleration, BI-2852 took 51.162 ps, and 5-Dehydroxyparatocarpin K (L1), Carpachromene (L2), Sanggenone H (L3), and Kuwanol C (L4) took 32.067 ps, 41.805 ps, 89.435 ps, and 44.557 ps, respectively. Notably, the Sanggenone H (L3) took 89.435 ps, nearly a two-fold increase when compared to the BI-2852. In addition, the L1, L2 and L4 were shown to be nearer to the BI-2852, with a 0.3-, 0.2- and 0.15-fold decrease, as shown in Table 6.

### 3.5. Molecular-Dynamics Simulation

**Figure 7:**
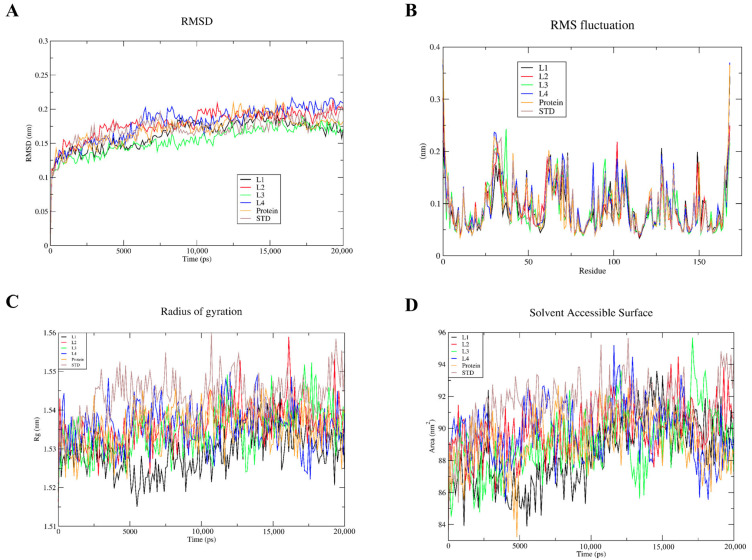
Root-mean-square deviation (RMSD) (**A**), root-mean-square fluctuation (RMSF) (**B**), radius of gyration (Rg) (**C**), and the solvent-accessible surface area (SASA) (**D**) of the docked complexes of BI-2852 (std) 5-Dehydroxyparatocarpin K (L1), Carpachromene (L2), Sanggenone H (L3), and Kuwanol C (L4), with the KRAS G12D mutant and KRAS G12D mutant alone (apoprotein).

The molecular dynamics simulation of BI-2852 and all the top four lead flavonoids with KRAS G12D was performed using YASARA, and the protein stability and conformations were predicted over a period of time. Root-mean-square deviation (RMSD), root- mean-square fluctuation (RMSF), radius of gyration (Rg), and the solvent-accessible surface area (SASA) of the docked complexes, along with the KRAS G12D protein alone (apoprotein) were calculated, and are shown in Figure 7. The RMSD plot shows that equilibrium was achieved at around 7500 ps (7.5 ns), and after that, all the protein–ligand complexes possess reasonably stable conformations. It is also observed that the RMSD range for all the ligands is within 0.12–0.22 nm, which confirms the stability of the docked complexes. The RMSF plot reveals that the fluctuations are less than 2 nm, which indicates that the docked complexes have greater stability. The fluctuations are seen at most of the loop regions of the 30–38, 58–74, 80–110, and 135–150 positions, which are far from the switch I and switch II regions of the KRAS G12D mutant. Even though there is a fluctuation observed at the switch-II (residues 57–76) region, the RMSF of that fluctuation is below 2 nm, showing that the docked complexes are stable. The Rg plot reveals that the KRAS G12D mutant protein is rigid around its axes, by showing Rg values ranging from 1.51 to 1.56 nm. All four lead flavonoids and BI-2852 attained stable nature from 5–9 ns (1.54 nm), and the lead 1 (5-Dehydroxyparatocarpin K) showed an Rg value comparatively lower than that of the BI-2852, while other lead flavonoids showed a similar type of stability nature as BI-2852. SASA is used as a measure of the water-accessible regions on the protein’s surface area, and thus a lower SASA value is indicated as a measure of the higher compactness of the protein complex. The SASA plot reveals that, for all the compounds having water-accessible regions on the protein’s surface area, the value range lies between 84 and 96 nm^2^. L2, L3, and L4 showed a similar range to BI-2852, while the L1 showed lower SASA than all the compounds. In combination, all four lead flavonoids showed similar ranges in the RMSD, RMSF, Rg, and SASA trajectories to BI-2852, and showed better stability of the docked complexes.

### 3.6. Lead-Flavonoid Effect on Other Molecular Targets

**Figure 8:**
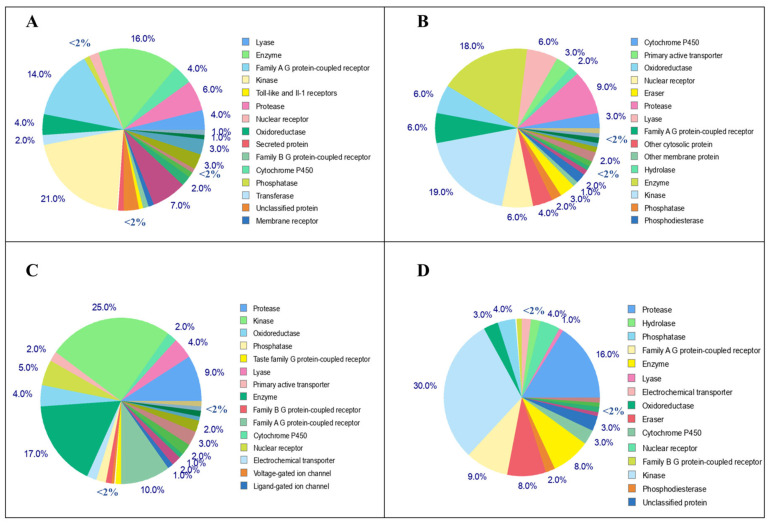
Molecular targets of top four lead flavonoids: the pie chart represents the predicted molecular targets such as kinase, cytosolic protein, lyase, oxidoreductase, protease, phosphatase, epigenetic erasers, cytochrome p450, GPCR (family A and B), nuclear receptor, ion channels, phosphodiesterase and membrane proteins of 5-Dehydroxyparatocarpin K (**A**), Carpachromene (**B**), Sanggenone H (**C**), and Kuwanol C (**D**).

From the above-mentioned studies, it is observed that the top four lead flavonoids have more binding affinities toward the KRAS G12D mutant protein. Furthermore, their effects against various molecular targets were predicted using the SwissTargetPrediction web server, and the data are shown in Figure 8. These four leads were reported to have effects on various molecular targets such as kinase, cytosolic protein, lyase, oxidoreductase, protease, phosphatase, epigenetic erasers, cytochrome p450, GPCR (family A and B), nuclear receptor, ion channels, phosphodiesterase, and membrane proteins. Notably, all four leads have a significant effect against the kinase targets, which also include the kinases involved in the MAPK (RAS/ERK/MEK) pathway. Comparatively, L4 has high possible effects against kinases as 30%, followed by L3 with 25%, L1 with 21%, and L2 with 19%, as shown in Figure 8. The complete list of the known kinase targets of all four leads was predicted, and is provided in Supplementary Table S2. These data also support the fact that these four leads could also possibly bind with the KRAS downstream effectors such as ERK and MEK kinases, and other cyclin-dependent kinases (CDKs) which regulate the cell cycle.

### 3.7. Toxicity and Cell-Line Cytotoxicity of Lead Flavonoids

The in silico cytotoxic potential of the top four lead flavonoids and BI-2852 against various cancer cell lines were predicted, based on Structure–activity relationship (SAR), using the CLC-Pred web server. The results were filtered by: (i) the probability of activeness (Pa) should be greater than the probability of inactiveness (Pi) (i.e, Pa > Pi), (ii) the probability of activeness (Pa) should be at least 30% (i.e, Pa > 0.3), and (iii) KRAS highly-expressed cancer types, such as pancreatic, lung, and colon. The data are provided in Table 7. The BI-2852, L1, L2, L3, and L4 were reported to have cytotoxic potential against the 2, 3, 2, 2, and 5 cancer cell lines, respectively. In addition, these compounds were reported to be active against small-cell lung carcinoma and non-small-cell lung carcinoma (NSCLC). Although these top four lead flavonoids have passed several tests, it is essential to analyze their toxicity to humans. Therefore, the toxicity of the top four lead flavonoids and BI-2852 were predicted, and the data provided in Table 8. The toxicity prediction reveals that all our four lead flavonoids, L1, L2, L3, and L4 have less toxicity than the BI-2852, which was confirmed by the values of H-HT (L2 and L3), DILI (L1 and L4), AMES (L2, L3 and L4), ROAT (L1, L2 and L4), and FDAMMD (L1, L2, L3 and L4) parameters. All four leads in comparison with BI-2852 showed excellent activity against NR-AR (L2, L3 and L4), NR-ER (L1, L2, L3 and L4), NR-PPAR-γ (L1, L2, L3 and L4), and SR-ARE (L1, L2, L3 and L4). Notably, the golden triangle was accepted for all four lead compounds, and the BI-2852 was rejected. Our data also strongly suggest that all four lead flavonoids possess less carcinogenicity when compared to the BI-2852, which might be due to the presence of fewer toxicophores, as shown in Table 8.

## 4. Discussion

Mutant KRAS plays a crucial role in proliferation and survival in several cancers, and G12, G13, and Q61 are the hotspot residues, with mutational frequencies of 83%, 14%, and 2%, respectively [13]. The G12D and G12C mutation frequencies are much higher, and were considered undruggable for the past 40 years, due to their smooth surface and lack of suitable pockets [42,43]. This came to an end with the discovery and FDA approval of the irreversible covalent KRAS G12C inhibitor, Sotorasib (AMG 510), for the treatment of NSCLC, in 2021 [15]. After this discovery, an adequate amount of studies have been carried out to discover more novel KRAS G12C inhibitors, and to efficiently drug the KRAS G12D, which has more mutation frequency than KRAS G12C and still lacks an inhibitor, due to the absence of reactive cysteine residue at the 12th position [16,17]. Accumulating pieces of evidence report that the KRAS G12C inhibitors such as Sotorasib (AMG 510), ARS-1620, and Adagrasib (MRTX849) are becoming resistant in non-small-cell-lung-cancer (NSCLC) and colorectal-cancer (CRC) patients [44,45]. Interestingly, recent reports suggest that targeting the switch I/II of KRAS G12D could be an effective strategy [18,19]. Alongside this, several reports suggest that flavonoids (polyphenols) possess a strong anticancer potential against various cancers, by inducing cell-cycle arrest at G0/G2 or G2/M, downregulation of p53, inhibition of tyrosine kinase and heat shock proteins, and inhibition of the expression of RAS-downstream-effector family proteins [46,47]. A Danish cohort study revealed that a 500 mg/day intake of flavonoids significantly reduces the risk of cancer incidence [48]. Flavonoids are also reported to have inhibitory activity against H-Ras mutants [49], NRAS mutants [50] and KRAS mutants [51,52].

Therefore, in the present study, we focused on targeting the switch I (residues 25–40) and switch II (residues 57–76) pocket as shown in Figure 2, and the amino-acid residues around 4 Å of this pocket were observed to be Lys5, Leu6, Val7, Glu37, Ser39, Asp54, Leu56, Gln70, Tyr71, Thr74, and Gly75. From a clear consideration of the recent reports on inhibitors raised against the KRAS G12D, we planned to screen the dietary bioflavonoids of plant origin through computational approaches. Initially, we retrieved the structural and molecular descriptors of nearly 1093 bioflavonoids with known biological activity, from PubChem and ChemFaces. Evaluation of drug-likeness and absorption and the distribution, metabolism, and excretion (ADME) properties of compounds plays a key role in the drug-discovery and development processes, by addressing safety and efficacy [31,32]. A total of 925 (duplicates removed) flavonoids were screened for their drug-likeness and ADME properties, using the SwissADME web server, which resulted in 514 flavonoids. Then, these 514 were selected for the molecular-docking analysis and for comparison with BI-2852 (reference). Mostly, all the flavonoids showed good binding affinities, with binding energies ranging from −8.8 Kcal/mol to −5 Kcal/mol, as shown in Supplementary Table S2. Although all the flavonoids showed good affinity with the KRAS G12D mutant, it is very important to show significant affinity towards the KRAS SI/SII pocket such as BI-2852, to yield the inhibition activity. These 514 bioflavonoids, along with the BI-2852, were docked at the same co-ordinates as the crystallized structure of BI-2852 with KRAS G12D (PBD ID: 6GJ8), which resulted in the identification of four potential lead flavonoids, namely 5-Dehydroxyparatocarpin K (L1), Carpachromene (L2), Sanggenone H (L3), and Kuwanol C (L4), with binding affinities of −8.8 Kcal/mol, −8.64 Kcal/mol, −8.62 Kcal/mol, and −8.58 Kcal/mol, respectively, while the BI-2852 showed −8.59 Kcal/mol against the KRAS G12D mutant protein. In addition, the drug-likeness properties and the ADME of these top four lead bioflavonoids are shown in Table 1 and Table 2, respectively.

The binding energies and the inhibitory constant (Ki) determines the binding affinity of a ligand molecule toward the protein [31]. The steered molecular dynamics predict the time taken to dissociate the ligand from the protein by providing an external pulling force, and measure the binding affinity of the protein–ligand complex [34]. Our results revealed that all our four leads showed excellent binding energies (in the range of −8.8 to −8.5) and Ki values (ranging from 0.83 to 28.54 µM), in comparison with the KRAS switch I/II inhibitor BI-2852, which showed a binding energy of −8.59 Kcal/mol and Ki value of 3.69 µM, as shown in Table 3. This might be due to the two or more H-bond interactions and other electrostatic and hydrophobic interactions of the lead four flavonoids against the KRAS G12D mutant, rather than the BI-2852, which only showed two H-bonds. Notably, from the SMD analysis the Sanggenone H (L3) took 89.435 ps, nearly a two-fold increase when compared to the BI-2852. In addition, the L1, L2 and L4 were shown to be nearer to the BI-2852, with a 0.3, 0.2 and 0.15-fold decrease, respectively, as shown in Table 6. It was observed that all our four lead flavonoids target and bind with the switch I/II regions of the KRAS G12D mutant when compared to the BI-2852 (reference), with more affinities, as shown in Figure 4.

From the molecular-dynamics simulations, the root-mean-square deviation (RMSD), root-mean-square fluctuation (RMSF), radius of gyration (Rg), and the solvent-accessible surface area (SASA) of the BI-2852 and lead flavonoids docked with the KRAS G12D mutant were predicted. The RMSD trajectory provides information about the protein stability upon ligand binding, RMSF provides information about the average mobility of the c-α atoms of amino-acid residues, Rg provides information about the folding and rigidity properties of the protein, while SASA provides information about the protein’s surface area available for water molecules [38,53]. In general, if the RMSD is lower, then the stability will be higher. The RMSD for all the BI-2852 and flavonoids were within the 0.12–0.22 nm range, which confirms the more stable nature of the docked complexes. From the RMSD analysis, it is observed that the L1, L2, L3, and L4 attained stability at 0.18 nm (10 ns), 0.2 nm (10 ns), 0.19 nm (13 ns), and 0.2 nm (8 ns),respectively, in comparison with BI-2852 at 0.18 nm (12 ns). The RMSF will be mostly observed higher in the loop and turns, and lower in the sheet and helix regions. RMSF plots of BI-2852 and the top four flavonoids have greater stability, showcased by fewer fluctuations (<0.2 nm). The fluctuations were mostly seen at the loop and turns, rather than the helix and sheet regions. Even though there is a slight fluctuation observed at the switch-II (residues 57–76) region, the range was observed to be below 2 nm, showing that the docked complexes are stable. Generally, the lower Rg values define the folding and rigidity properties of the protein complex. All four lead flavonoids and BI-2852 attained stable nature from 5–9 ns (1.54 nm). Comparatively, 5-Dehydroxyparatocarpin K (L1) showed a lower Rg value than the BI-2852, and attained stability at 8 ns (1.53 nm). Essentially, SASA is used as a measure of the water-accessible regions on the protein’s surface area. If the SASA value is higher, then the protein complex is more flexible, and if the SASA value is lower, then the protein complex is more compact. The SASA value of all the flavonoids and BI-2852 lies between 84–96 nm^2^, indicating the compactness of the protein complexes. Comparatively, the L1 showed a lower SASA value than the BI-2852, indicating its more compact nature. In addition, the MD simulations of apoprotein (KRAS G12D mutant-protein alone) show fewer fluctuations and good stability. Overall, all four lead flavonoids showed similar ranges for the RMSD, RMSF, Rg, and SASA trajectories of BI-2852, and showed better stability of the docked complexes, as shown in Figure 7.

**Figure 9:**
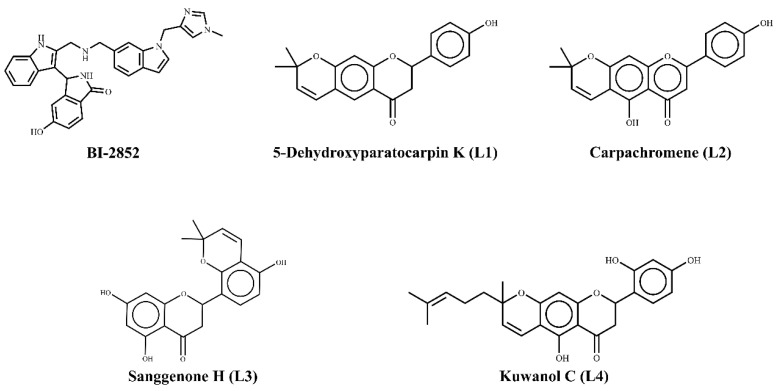
2D structures of BI-2852 and top 4 lead flavonoids.

SwissTargetPrediction, which uses the similarity principle of two compounds possessing similar properties, was utilized to predict the other possible kinase targets of the lead compounds. These four leads were reported to have effects on various molecular targets such as kinase, cytosolic protein, lyase, oxidoreductase, protease, phosphatase, epigenetic erasers, cyto-chrome p450, GPCR (family A and B), nuclear receptor, ion channels, phosphodiesterase, and membrane proteins [54,55,56,57]. The 2D structures of the 5-Dehydroxyparatocarpin K (L1), Carpachromene (L2), Sanggenone H (L3), and Kuwanol C (L4,) along with the BI-2852 (reference), are shown in the Figure 9.

The toxicological evaluation of the lead compounds was used to evaluate the correlation between the therapeutic dose and the toxic dose, and is used to predict the toxic dosage of any compound in the drug-discovery processes [58]. Our compounds were predicted to be less toxic than the BI-2852, as predicted by the CLC-Pred web server. The toxicity prediction reveals that all our four lead flavonoids, L1, L2, L3, and L4 have less toxicity than the BI-2852, which was confirmed by the values of H-HT (L2 and L3), DILI (L1 and L4), AMES (L2, L3 and L4), ROAT (L1, L2 and L4), and FDAMMD (L1, L2, L3 and L4) parameters. All four leads, in comparison with BI-2852, showed excellent activity against NR-AR (L2, L3 and L4), NR-ER (L1, L2, L3 and L4), NR-PPAR-γ (L1, L2, L3 and L4), and SR-ARE (L1, L2, L3 and L4), and were less carcinogenic, which might be due to the presence of fewer toxicophores, as shown in Table 8. Notably, the golden triangle was accepted for all four lead compounds, and the BI-2852 was rejected. In addition, the in silico cell-line-toxicity prediction suggests that our four leads could possess cytotoxicity against the pancreatic, lung, and colon cancers in which the KRAS is highly mutated [42,43]. Inhibition of KRAS G12D mutant’s downstream effectors could also be an effective strategy against the KRAS-mutated solid tumors [59,60,61]. Our analysis also showed that these top four lead flavonoids could also be effective against KRAS downstream effectors such as ERK and MEK kinases, and other cyclin-dependent kinases (CDKs) which regulate the cell cycle, as shown in the Supplementary Table S2.

## 5. Conclusions

The current study attempted to evaluate the KRAS G12D inhibition potential of dietary bioflavonoids through various computational studies. Herein, we have evaluated the drug-likeness properties, ADME properties, binding affinities, steered molecular dynamics, the possible activity against kinases, toxicity, and the in silico cancer-cell-line-cytotoxicity profiles of the flavonoids. This study yielded the identification of four potential lead flavonoids, namely 5-Dehydroxyparatocarpin K (L1), Carpachromene (L2), Sanggenone H (L3), and Kuwanol C (L4) as potential lead inhibitors of the KRAS G12D mutant, with binding affinities of −8.8 Kcal/mol, −8.6 Kcal/mol, −8.6 Kcal/mol, and −8.5 Kcal/mol respectively, in comparison with BI-2852 (−8.5 Kcal/mol), and also possessing similar stability to BI-2852 in molecular-dynamics simulations. Interestingly, these four lead flavonoids showed more inhibitory activity than BI-2852 against KRAS G12D, via the targeting of the SI/II regions. From our findings, we conclude that these 4 flavonoids potentially inhibit KRAS G12D mutants with high binding affinities. However, it is confirmed by the computational studies that these compounds have to be studied in vitro and in vivo to evaluate their complete therapeutic potential against the KRAS G12D mutant cancers. We also suggest utilizing these four flavonoids in the development of antibody-drug conjugates (ADCs) and proteolysis-targeting-chimera (PROTAC) technologies for the effective treatment of KRAS G12D mutated cancers in the near future.

## Figures and Tables

**Table 1:** Physicochemical properties of 4 lead flavonoids.

Flavonoid/Compound Name	Physicochemical Properties								
	MF	MW	HA	AHA	HBD	HBA	RB	MR	TPSA
BI-2852 (reference)	C_31_H_28_N_6_O_2_	516.59	39	29	4	4	7	155.12	99.9
5-Dehydroxy paratocarpin K (L1)	C_20_H_18_O_4_	322.35	24	12	1	4	1	91.65	55.76
Carpachromene (L2)	C_20_H_16_O_5_	336.34	25	16	2	5	1	96.09	79.9
Sanggenone H (L3)	C_20_H_18_O_6_	354.35	26	12	3	6	1	95.69	96.22
Kuwanol C (L4)	C_25_H_26_O_6_	422.47	31	12	3	6	4	119.25	96.22

Note: MF—Molecular weight; MW-Molecular weight in g/mol; HA—Heavy atoms; AHA—Aromatic heavy atoms; HBD—Hydrogen-bond donor; HBA—Hydrogen-bond acceptor; RB—Rotatable bonds; MR—Molar refractivity in m^3^/mol; TPSA—Topological polar surface area in Å.

**Table 2:** Lipophilicity, hydrophilicity, pharmacokinetics, drug-likeness and medicinal-chemistry properties of 4 lead flavonoids.

Flavonoid/Compound Name	Lipophilicity	Water Solubility	Pharmacokinetics		Drug-Likeness		Medicinal Chemistry
	Consensus Log Po/w	LogS	GIA	BBBP	Lipinski	BA	SA
BI-2852 (reference)	3.2	−4.98	High	No	No	0.55	4.25
5-Dehydroxy paratocarpin K (L1)	3.31	−4.33	High	Yes	Yes	0.55	3.92
Carpachromene (L2)	3.32	−4.82	High	No	Yes	0.55	3.71
Sanggenone H (L3)	2.71	−4.48	High	No	Yes	0.55	4.03
Kuwanol C (L4)	4.15	−5.8	High	No	Yes	0.55	4.79

Note: Consensus Log Po/w—Average of all the five predictions of lipophilicity—Log Po/w (iLOGP), Log Po/w (XLOGP3), Log Po/w (WLOGP), Log Po/w (MLOGP), Log Po/w (SILICOS-IT); LogS—water solubility of a drug; GIA—Gastro-Intestinal absorption; BBBP—Blood–brain barrier permeability; BA—Bioavailability; SA—Synthetic accessibility from 1–10 (very easy–very difficult).

**Table 3:** Binding energies, inhibitory constant, and interacting residues of 4 leads.

Compound Name	Binding Energy (Kcal/mol)	Ki (µM)	Interacting Residues
BI-2852	−8.59	3.69	Lys5, Leu6, Val7, Glu37, Ser39, Asp54, Leu56, Cln70, Tyr71, Thr74, Gly75
L1	−8.80	4.19	Lys5, Leu6, Val7, Glu37, Asp54, Leu56, Met67, Gln70, Tyr71, Thr74
L2	−8.64	7.95	Lys5, Leu6, Val7, Glu37, Asp54, Leu56, Met67, Gln70, Tyr71, Thr74, Gly75
L3	−8.62	0.83	Lys5, Leu6, Val7, Glu37, Ser39, Asp54, Leu56, Met67, Gln70, Tyr71, Thr74, Gly75
L4	−8.58	28.54	Lys5, Leu6, Val7, Glu37, Asp54, Ile55, Leu56, Met67, Gln70, Tyr71, Thr74, Gly75

**Table 4:** Intermolecular hydrogen-bond interactions of 4 leads.

Flavonoid Name	Interaction	Distance (Å)
BI-2852 (reference)	OG atom in Ser39 with N2 atom in BI-2852	3.11
	OD1 atom in Asp54 with N1 atom in BI-2852	2.84
5-Dehydroxyparatocarpin K (L1)	O atom in Leu6 with O4 atom in L1	2.79
	O atom in Asp54 with O4 atom in L1	3.00
Carpachromene (L2)	O atom in Leu6 with O4 atom in L2	2.78
	O atom in Asp54 with O4 atom in L2	2.97
Sanggenone H (L3)	O atom in Asp54 with O4 atom in L3	3.19
Kuwanol C (L4)	O atom in Leu6 with O5 atom in L4	2.70
	O atom in Asp54 with O5 atom in L4	2.48
	O atom in Tyr71 with O4 atom in L4	2.70

**Table 5:** Intermolecular electrostatic and hydrophobic interactions of 4 lead flavonoids.

Interactions	BI-2852	L1	L2	L3	L4
Electrostatic					
Charge	Asp54	**-**	**-**	**-**	**-**
Hydrophobic					
Alkyl	-	Met67	Met67	Leu56	Met67
Mixed Pi-Alkyl	Leu56	Lys5, Leu56	Lys5, Leu56	Lys5, Leu56	Lys5, Leu56

**Table 6:** Steered molecular dynamics of 4 leads.

Flavonoid	Time Taken to Dissociate the Ligand (ps)
BI-2852 (reference)	51.162
L1	32.067
L2	41.805
L3	89.435
L4	44.557

**Table 7:** Cancer-cell-line cytotoxicity prediction of top 4 lead flavonoids.

Flavonoids/Compounds	Pa	Pi	Cell Line	Type	Region
BI-2852	0.356	0.218	DMS-114	Lung carcinoma	Lung
	0.326	0.041	SW-620	Colon adenocarcinoma	Colon
L1	0.457	0.029	NCI-H187	Small-cell lung carcinoma	Lung
	0.421	0.024	HOP-18	Non-small-cell lung carcinoma	Lung
	0.365	0.053	PC-6	Small-cell lung carcinoma	Lung
L2	0.430	0.022	HOP-18	Non-small-cell lung carcinoma	Lung
	0.395	0.075	NCI-H187	Small-cell lung carcinoma	Lung
L3	0.476	0.020	NCI-H187	Small-cell lung carcinoma	Lung
	0.459	0.017	HOP-18	Non-small-cell lung carcinoma	Lung
L4	0.604	0.004	HOP-18	Non-small-cell lung carcinoma	Lung
	0.470	0.023	NCI-H187	Small-cell lung carcinoma	Lung
	0.369	0.056	NCI-H322M	Non-small-cell lung carcinoma	Lung
	0.360	0.042	NCI-H522	Non-small-cell lung carcinoma	Lung
	0.314	0.077	NCI-H226	Non-small-cell lung carcinoma	Lung

**Table 8:** Toxicity profiles of top 4 lead flavonoids.

Toxicity	BI-2852	L1	L2	L3	L4
H-HT	0.846	0.847	0.372	0.275	0.851
DILI	0.953	0.484	0.941	0.905	0.806
AMES	0.256	0.409	0.195	0.108	0.086
ROAT	0.75	0.189	0.193	0.883	0.749
FDAMMD	0.978	0.671	0.848	0.907	0.905
NR-AR	0.016	0.041	0.005	0.005	0.006
NR-ER	0.215	0.804	0.938	0.625	0.666
NR-PPAR-γ	0.13	0.807	0.984	0.974	0.972
SR-ARE	0.73	0.915	0.939	0.941	0.951
Carcinogenicity	0.088	0.875	0.79	0.71	0.76
Toxicophores	4	1	1	1	1
Golden Triangle	Rejected	Accepted	Accepted	Accepted	Accepted

Note: 0–0.3 (poor activity); 0.3–0.7 (medium activity); and 0.7–1.0 (high activity).

## Data Availability

Not applicable.

## Supplementary Materials

The following supporting information can be downloaded at https://www.mdpi.com/article/10.3390/cimb45030137/s1, Table S1: Binding energies of all 514 bioflavonoids with the KRAS G12D mutant protein; Table S2: Kinase targets of top 4 lead flavonoids; Figure S1: Binding poses and molecular interactions of BI-2852 in crystallized and docked forms; Figure S2: Binding energies of BI-2852 and top 4 lead flavonoids against KRAS G12D mutant protein.
