# Supplementary material for: Identification of Dietary Bioflavonoids as Potential Inhibitors against KRAS G12D Mutant—Novel Insights from Computer-Aided Drug Discovery
Source: Curr Issues Mol Biol. 2023 Mar 6;45(3):2136–56. doi: 10.3390/cimb45030137 (PMC10047893; doi:10.3390/cimb45030137)
Supplement: Supplementary file 1 [file cimb-45-00137-s001.zip › cimb-2175797-supplementary.pdf]

Supplementary materials

Identification of Dietary Bioflavonoids as Potential Inhibitors against KRAS G12D Mutant—Novel Insights from Computer-Aided Drug Discovery

Prasanna Srinivasan Ramalingam [1,2,†], Purushothaman Balakrishnan [2,†], Senthilnathan Rajendran [3], Arunachalam Jothi [3], Rajasekaran Ramalingam [4] and Sivakumar Arumugam [1,*]

1 Protein Engineering lab, School of Biosciences and Technology, VIT University, Vellore 632014, Tamil Nadu, India
2 TanBio R and D Solution, Thanjavur 613403, Tamil Nadu, India
3 Department of Bioinformatics, School of Chemical and Biotechnology, SASTRA Deemed University, Thanjavur 613401, Tamil Nadu, India
4 Quantitative Biology Lab, School of Biosciences and Technology, VIT University, Vellore 632014, Tamil Nadu, India
* Correspondence: siva_kumar.a@vit.ac.in
† These authors contributed equally to this work.

Contents:

Supplementary Table S1: Binding energies of all 514 bioflavonoids with the KRAS G12D mutant protein.

Supplementary Table S2: Kinase targets of top 4 lead flavonoids.

Supplementary Figure S1: Binding poses and molecular interactions of BI-2852 in crystallized and docked forms.

Supplementary Figure S2: Binding energies of BI-2852 and top 4 lead flavonoids against KRAS G12D mutant protein.

Supplementary Table S1: Binding energies of all 514 bioflavonoids with the KRAS G12D mutant protein. The binding energy (Kcal/mol) of all the compounds were tabulated according to their higher negative values and the BI-2852 was shown in bold.

Ligand model number	Flavonoid/ Compound name	Canonical SMILES	Binding Affinity
model_684	5-Dehydroxyparatocarpin K	Oc1ccc(cc1)C1CC(=O)c2c(O1)cc1c(c2)C=CC(O1)(C)C	-8.8
model_298	Carpachromene	Oc1ccc(cc1)c1cc(=O)c2c(o1)cc1c(c2O)C=CC(O1)(C)C	-8.6
model_765	Sanggenone H	Oc1cc2OC(CC(=O)c2c(c1)O)c1ccc(c2c1OC(C)(C)C=C2)O	-8.6
model_654	Kuwanol C	CC(=CCCC1(C)C=Cc2c(O1)cc1c(c2O)C(=O)CC(O1)c1ccc(c c1O)O)C	-8.5
**Reference**	**BI-2852**	**CN1C=C(N=C1)CN2C=CC3=C2C=C(C=C3)CNCC4=C(C 5=CC=CC=C5N4)C6C7=C(C=CC(=C7)O)C(=O)N6**	**-8.5**
model_379	Kazinol B	CC(=CCc1c(cc2c(c1O)OC(C=C2)(C)C)C1CCc2c(O1)cc(cc2) O)C	-8.4
model_395	Cyclomorusin	CC(=CC1Oc2cc(O)ccc2c2c1c(=O)c1c(o2)c2C=CC(Oc2cc1O) (C)C)C	-8.4
model_874	Furano(2",3",7,6)-4'-hydroxyflavanone	Oc1ccc(cc1)C1CC(=O)c2c(O1)cc1c(c2)cco1	-8.4
model_138	Barbacarpan	CC(=C)C1Oc2c(C1)c1OC3C(c1cc2)COc1c3ccc(c1)O	-8.3
model_816	Licoisoflavone B	Oc1cc(O)c2c(c1)occ(c2=O)c1ccc2c(c1O)C=CC(O2)(C)C	-8.3
model_100	Maackiain	Oc1ccc2c(c1)OCC1C2Oc2c1cc1c(c2)OCO1	-8.2
model_799	Ophiopogonanone A	O=C1C(COc2c1c(O)c(c(c2)O)C)Cc1ccc2c(c1)OCO2	-8.2
model_92	Glabridin	Oc1ccc(c(c1)O)C1COc2c(C1)ccc1c2C=CC(O1)(C)C	-8.2
model_970	Cudraflavone B	CC(=CCc1c(oc2c(c1=O)c(O)c1c(c2)OC(C=C1)(C)C)c1ccc(c c1O)O)C	-8.2
model_814	Glabrene	Oc1ccc2c(c1)OCC(=C2)c1ccc(c2c1OC(C)(C)C=C2)O	-8.1
model_257	Methylophiopogonanone A	O=C1C(COc2c1c(O)c(C)c(c2C)O)Cc1ccc2c(c1)OCO2	-8
model_400	Butin	Oc1ccc2c(c1)OC(CC2=O)c1ccc(c(c1)O)O	-8
model_689	Methylophiopogonone A	Cc1c(O)c(C)c2c(c1O)c(=O)c(co2)Cc1ccc2c(c1)OCO2	-8
model_758	Semilicoisoflavone B	Oc1cc(O)c2c(c1)occ(c2=O)c1cc(O)c2c(c1)C=CC(O2)(C)C	-8
model_785	7,8-Benzoflavone	O=c1cc(oc2c1ccc1c2cccc1)c1ccccc1	-8
model_1002	Methylophiopogonone B	COc1ccc(cc1)Cc1coc2c(c1=O)c(O)c(c(c2C)O)C	-7.9
model_1056	Ophiopogonanone B	COc1ccc(cc1)CC1COc2c(C1=O)c(O)c(c(c2)O)C	-7.9
model_128	Bavachin	CC(=CCc1cc2C(=O)CC(Oc2cc1O)c1ccc(cc1)O)C	-7.9
model_134	Fustin	Oc1ccc2c(c1)OC(C(C2=O)O)c1ccc(c(c1)O)O	-7.9

model_258	Methylophiopogonanone B	COc1ccc(cc1)CC1COc2c(C1=O)c(O)c(c(c2C)O)C	-7.9
model_286	Corylin	Oc1ccc2c(c1)occ(c2=O)c1ccc2c(c1)C=CC(O2)(C)C	-7.9
model_49	Liquiritigenin	Oc1ccc(cc1)C1CC(=O)c2c(O1)cc(cc2)O	-7.9
model_581	7-Hydroxy-3-(4-hydroxybenzyl)chromic	Oc1ccc(cc1)CC1COc2c(C1)ccc(c2)O	-7.9
model_604	Isoderrone	Oc1cc(O)c2c(c1)occ(c2=O)c1ccc2c(c1)C=CC(O2)(C)C	-7.9
model_624	Cedeodarin	Oc1ccc(cc1O)C1Oc2cc(O)c(c(c2C(=O)C1O)O)C	-7.9
model_839	Poriol	Oc1ccc(cc1)C1CC(=O)c2c(O1)cc(c(c2O)C)O	-7.9
model_1038	Pomiferin	CC(=CCc1c2OC(C)(C)C=Cc2c2c(c1O)c(=O)c(co2)c1ccc(c(c1)O)O)C	-7.8
model_1060	Licoflavone A	CC(=CCc1cc2c(=O)cc(oc2cc1O)c1ccc(cc1)O)C	-7.8
model_1066	Glyasperin F	Oc1cc(O)c2c(c1)OCC(C2=O)c1ccc(c2c1OC(C)(C)C=C2)O	-7.8
model_118	Dihydromorin	Oc1ccc(c(c1)O)C1Oc2cc(O)cc(c2C(=O)C1O)O	-7.8
model_169	Fisetin	Oc1ccc2c(c1)oc(c(c2=O)O)c1ccc(c(c1)O)O	-7.8
model_17	3-Deoxysappanone B	Oc1ccc2c(c1)OCC(C2=O)Cc1ccc(c(c1)O)O	-7.8
model_218	Anhydrotuberosin	Oc1ccc2c(c1)OCc1c2oc2c1cc1c(c2)OC(C=C1)(C)C	-7.8
model_266	7-O-Methyleriodictyol	COc1cc2OC(CC(=O)c2c(c1)O)c1ccc(c(c1)O)O	-7.8
model_280	Scillascillin	Oc1cc(O)c2c(c1)OCC1(C2=O)Cc2c1cc1c(c2)OCO1	-7.8
model_304	Isobonducellin	COc1ccc(cc1)C=C1COc2c(C1=O)ccc(c2)O	-7.8
model_726	Demethylvestitol	Oc1ccc2c(c1)OCC(C2)c1ccc(cc1O)O	-7.8
model_768	Coccineone B	Oc1cc(O)c2c(c1)oc1c(c2=O)c2ccccc2OC1O	-7.8
model_788	4'-Hydroxyflavanone	Oc1ccc(cc1)C1CC(=O)c2c(O1)cccc2	-7.8
model_833	Plathymenin	O=C1CC(Oc2c1cc(O)c(c2)O)c1ccc(c(c1)O)O	-7.8
model_834	Garbanzol	Oc1ccc(cc1)C1Oc2cc(O)ccc2C(=O)C1O	-7.8
model_87	Eriodictyol	Oc1cc2OC(CC(=O)c2c(c1)O)c1ccc(c(c1)O)O	-7.8
model_1036	Glabrone	Oc1ccc2c(c1)occ(c2=O)c1ccc2c(c1O)C=CC(O2)(C)C	-7.7
model_1062	Licoflavone B	CC(=CCc1cc2c(=O)cc(oc2cc1O)c1ccc(c(c1)CC=C(C)C)O)C	-7.7
model_121	Morin	Oc1ccc(c(c1)O)c1oc2cc(O)cc(c2c(=O)c1O)O	-7.7
model_167	Sakuranetin	COc1cc2OC(CC(=O)c2c(c1)O)c1ccc(cc1)O	-7.7
model_188	Alpinumisoflavone	Oc1ccc(cc1)c1coc2c(c1=O)c(O)c1c(c2)OC(C=C1)(C)C	-7.7
model_206	7,4'-Dihydroxy-3'-prenylflavan	CC(=CCc1cc(ccc1O)C1CCc2c(O1)cc(cc2)O)C	-7.7
model_231	Taxifolin	Oc1cc2OC(c3ccc(c(c3)O)O)C(C(=O)c2c(c1)O)O	-7.7
model_239	Naringenin	Oc1ccc(cc1)C1CC(=O)c2c(O1)cc(cc2O)O	-7.7
model_253	Luteolin	Oc1cc(O)c2c(c1)oc(cc2=O)c1ccc(c(c1)O)O	-7.7

model_292	Steppogenin	Oc1ccc(c(c1)O)C1CC(=O)c2c(O1)cc(cc2O)O	-7.7
model_388	Derrone	Oc1ccc(cc1)c1coc2c(c1=O)c(O)cc1c2C=CC(O1)(C)C	-7.7
model_411	7,3',4'-Trihydroxyflavone	Oc1ccc(cc1)c1oc2cc(O)ccc2c(=O)c1O	-7.7
model_578	Euchrenone A10	CC(=CCc1c(O)cc(c2c1OC(CC2=O)c1ccc2c(c1)C=CC(O2)(C)C)O)C	-7.7
model_626	Osajin	CC(=CCc1c2OC(C)(C)C=Cc2c2c(c1O)c(=O)c(co2)c1ccc(cc1)O)C	-7.7
model_644	Atalantoflavone	Oc1ccc(cc1)c1cc(=O)c2c(o1)c1C=CC(Oc1cc2O)(C)C	-7.7
model_697	6-Aldehydoisoophiopogonanone A	O=Cc1c(O)c2C(=O)C(COc2c(c1O)C)Cc1ccc2c(c1)OCO2	-7.7
model_773	Isookanin	O=C1CC(Oc2c1ccc(c2O)O)c1ccc(c(c1)O)O	-7.7
model_796	Ophiopogonanone C	O=Cc1c2OCC(C(=O)c2c(c(c1O)C)O)Cc1ccc2c(c1)OCO2	-7.7
model_971	Shuterin	CC(=CCc1c(O)cc2c(c1O)C(=O)C(C(O2)c1ccc(cc1)O)O)C	-7.7
model_975	Hyperxanthone E	Oc1cc(O)c2c(c1)oc1c(c2=O)c2CCC(Oc2c(c1)O)(C)C	-7.7
model_13	7,3',4'-Trihydroxy-3-benzyl-2H-chromene	Oc1ccc2c(c1)OCC(=C2)Cc1ccc(c(c1)O)O	-7.6
model_130	Hydroxygenkwanin	COc1cc(O)c2c(c1)oc(cc2=O)c1ccc(c(c1)O)O	-7.6
model_222	Genkwanin	COc1cc(O)c2c(c1)oc(cc2=O)c1ccc(cc1)O	-7.6
model_23	Quercetin	Oc1cc(O)c2c(c1)oc(c(c2=O)O)c1ccc(c(c1)O)O	-7.6
model_233	Aromadendrin	Oc1ccc(cc1)C1Oc2cc(O)cc(c2C(=O)C1O)O	-7.6
model_242	Hydrangenol	Oc1ccc(cc1)C1OC(=O)c2c(C1)cccc2O	-7.6
model_267	Kaempferol	Oc1ccc(cc1)c1oc2cc(O)cc(c2c(=O)c1O)O	-7.6
model_272	Apigenin	Oc1ccc(cc1)c1cc(=O)c2c(o1)cc(cc2O)O	-7.6
model_306	5-O-Methylnaringenin	COc1cc(O)cc2c1C(=O)CC(O2)c1ccc(cc1)O	-7.6
model_327	Isowighteone	CC(=CCc1cc(ccc1O)c1coc2c(c1=O)c(O)cc(c2)O)C	-7.6
model_340	Padmatin	COc1cc2OC(c3ccc(c(c3)O)O)C(C(=O)c2c(c1)O)O	-7.6
model_382	Hydroxytuberosone	O=C1C=CC2(C(=C1)OCC1(C2Oc2c1cc1c(c2)OC(C=C1)(C)C)O)O	-7.6
model_444	6-Prenylnaringenin	CC(=CCc1c(O)cc2c(c1O)C(=O)CC(O2)c1ccc(cc1)O)C	-7.6
model_525	Albanin A	CC(=CCc1c(oc2c(c1=O)c(O)cc(c2)O)c1ccc(cc1O)O)C	-7.6
model_526	beta-Rhamnocitrin	COc1cc(O)c2c(c1)oc(c(c2=O)O)c1ccc(c(c1)O)O	-7.6
model_591	Sanggenol A	CC(=CCc1c(O)ccc(c1O)C1CC(=O)c2c(O1)cc(cc2O)O)CCC=C(C)C	-7.6
model_605	Yukovanol	Oc1ccc(cc1)C1Oc2c(C(=O)C1O)c(O)cc1c2C=CC(O1)(C)C	-7.6
model_610	Dihydrobonducellin	COc1ccc(cc1)CC1COc2c(C1=O)ccc(c2)O	-7.6
model_637	2-Hydroxynaringenin	Oc1ccc(cc1)C1(O)CC(=O)c2c(O1)cc(cc2O)O	-7.6

model_668	Dihydroobovatin	O=C1CC(Oc2c1c(O)cc1c2CCC(O1)(C)C)c1ccccc1	-7.6
model_798	6-Aldehydo-isoophiopogonone A	O=Cc1c(O)c(C)c2c(c1O)c(=O)c(co2)Cc1ccc2c(c1)OCO2	-7.6
model_856	3,9-Dihydroeucomin	COc1ccc(cc1)CC1COc2c(C1=O)c(O)cc(c2)O	-7.6
model_890	4-O-Methylbutein	COc1ccc(cc1O)C=CC(=O)c1ccc(cc1O)O	-7.6
model_923	Eucomol	COc1ccc(cc1)CC1(O)COc2c(C1=O)c(O)cc(c2)O	-7.6
model_973	Gericudranin E	Oc1ccc(cc1)Cc1c(O)cc2c(c1O)C(=O)C(C(O2)c1ccc(cc1)O)O	-7.6
model_974	Artocarpesin	CC(=CCc1c(O)cc2c(c1O)c(=O)cc(o2)c1ccc(cc1O)O)C	-7.6
model_988	5,7-Dimethoxyluteolin	COc1cc(OC)c2c(c1)oc(cc2=O)c1ccc(c(c1)O)O	-7.6
model_1011	Azaleatin	COc1cc(O)cc2c1c(=O)c(c(o2)c1ccc(c(c1)O)O)O	-7.5
model_1083	Tuberosin	Oc1ccc2c(c1)OCC1(C2Oc2c1cc1c(c2)OC(C=C1)(C)C)O	-7.5
model_127	Bavachinin	COc1cc2OC(CC(=O)c2cc1CC=C(C)C)c1ccc(cc1)O	-7.5
model_139	Agrimonolide	COc1ccc(cc1)CCC1OC(=O)c2c(C1)cc(cc2O)O	-7.5
model_153	(-)-Epiafzelechin	Oc1ccc(cc1)C1Oc2cc(O)cc(c2CC1O)O	-7.5
model_210	Scutellarein	Oc1ccc(cc1)c1cc(=O)c2c(o1)cc(c(c2O)O)O	-7.5
model_250	Epicatechin	Oc1cc2OC(c3ccc(c(c3)O)O)C(Cc2c(c1)O)O	-7.5
model_311	Morusin	CC(=CCc1c(oc2c(c1=O)c(O)cc1c2C=CC(O1)(C)C)c1ccc(cc1 O)O)C	-7.5
model_316	Dihydroalpinumisoflavone	Oc1ccc(cc1)c1coc2c(c1=O)c(O)c1c(c2)OC(CC1)(C)C	-7.5
model_320	4',5-Dihydroxyflavone	Oc1ccc(cc1)c1cc(=O)c2c(o1)cccc2O	-7.5
model_326	4',5,7-Trihydroxy-6-prenylflavone/ 6-Prenylapigenin	CC(=CCc1c(O)cc2c(c1O)c(=O)cc(o2)c1ccc(cc1)O)C	-7.5
model_363	6-Methoxynaringenin	COc1c(O)cc2c(c1O)C(=O)CC(O2)c1ccc(cc1)O	-7.5
model_381	8-Isomulberrin hydrate	Oc1ccc(c(c1)O)c1oc2c3CCC(Oc3cc(c2c(=O)c1CCC(O)(C)C) O)(C)C	-7.5
model_399	Rhamnocitrin	COc1cc(O)c2c(c1)oc(c(c2=O)O)c1ccc(cc1)O	-7.5
model_487	8-Demethylsideroxylin	COc1cc2oc(cc(=O)c2c(c1C)O)c1ccc(cc1)O	-7.5
model_506	Isoneobavaisoflavone	Oc1ccc2c(c1)occ(c2=O)c1ccc2c(c1)CCC(O2)(C)C	-7.5
model_509	Erythrinin A	Oc1ccc(cc1)c1coc2c(c1=O)cc1c(c2)OC(C=C1)(C)C	-7.5
model_538	6-Hydroxykaempferol	Oc1ccc(cc1)c1oc2cc(O)c(c(c2c(=O)c1O)O)O	-7.5
model_553	Cyclomulberrin	CC(=CCc1c(O)cc(c2c1oc1c3ccc(cc3OC(c1c2=O)C=C(C)C)O)O)C	-7.5
model_556	5,7-Di-O-methylquercetin	COc1cc(O)c2c(c1)oc(c(c2=O)OC)c1ccc(c(c1)O)O	-7.5
model_560	Kaempferol 5-methyl ether	COc1cc(O)cc2c1c(=O)c(c(o2)c1ccc(cc1)O)O	-7.5
model_563	Cudraflavanone B	CC(=CCc1c(O)cc2c(c1O)C(=O)CC(O2)c1ccc(cc1O)O)C	-7.5
model_568	Eriosematin A	CC(=CCc1c(O)cc(c2c1occc2=O)O)C	-7.5

model_593	6-Methoxyluteolin	COc1c(O)cc2c(c1O)c(=O)cc(o2)c1ccc(c(c1)O)O	-7.5
model_603	Sanggenol L	CC(=CCCC1(C)C=Cc2c(O1)cc(c1c2OC(CC1=O)c1ccc(cc1O)O)O)C	-7.5
model_628	Neorauflavane	COc1c2CC(COc2cc2c1C=CC(O2)(C)C)c1ccc(cc1O)O	-7.5
model_643	Neorauflavene	COc1c2C=C(COc2cc2c1C=CC(O2)(C)C)c1ccc(cc1O)O	-7.5
model_656	7-O-Methylporiol	COc1cc2OC(CC(=O)c2c(c1C)O)c1ccc(cc1)O	-7.5
model_676	Isokaempferide	COc1c(oc2c(c1=O)c(O)cc(c2)O)c1ccc(cc1)O	-7.5
model_696	4',7-Isoflavandiol	Oc1ccc(cc1)C1COc2c(C1)ccc(c2)O	-7.5
model_704	Thevetiaflavone	COc1cc(O)cc2c1c(=O)cc(o2)c1ccc(cc1)O	-7.5
model_73	Thunberginol C	Oc1ccc(cc1)C1OC(=O)c2c(C1)cc(cc2O)O	-7.5
model_76	3-O-Methylquercetin	COc1c(oc2c(c1=O)c(O)cc(c2)O)c1ccc(c(c1)O)O	-7.5
model_763	Kuwanon A	CC(=CCc1c(oc2c(c1=O)c(O)cc(c2)O)c1ccc(c2c1OC(C)(C)C=C2)O)C	-7.5
model_850	Sophoraisoflavone A	Oc1cc(O)c2c(c1)occ(c2=O)c1ccc(c2c1OC(C)(C)C=C2)O	-7.5
model_857	Topazolin	COc1c(oc2c(c1=O)c(O)c(c(c2)O)CC=C(C)C)c1ccc(cc1)O	-7.5
model_9	3'-Hydroxy-3,9-dihydroeucomin	COc1ccc(cc1O)CC1COc2c(C1=O)c(O)cc(c2)O	-7.5
model_932	Licoflavonol	CC(=CCc1c(O)cc2c(c1O)c(=O)c(c(o2)c1ccc(cc1)O)O)C	-7.5
model_96	Cyanidin Chloride	Oc1cc(O)c2c(c1)[o+]c(c(c2)O)c1ccc(c(c1)O)O.[Cl-]	-7.5
model_969	Mesopsin	Oc1ccc(cc1)CC1(O)Oc2c(C1=O)c(O)cc(c2)O	-7.5
model_10	4-Demethyl-3,9-dihydroeucomin	Oc1ccc(cc1)CC1COc2c(C1=O)c(O)cc(c2)O	-7.4
model_1055	Cochliophilin A	O=c1cc(oc2c1c(O)c1c(c2)OCO1)c1ccccc1	-7.4
model_211	3,4,4',7-Tetrahydroxyflavan	Oc1ccc(cc1)C1Oc2cc(O)ccc2C(C1O)O	-7.4
model_219	Luteone	CC(=CCc1c(O)cc2c(c1O)c(=O)c(co2)c1ccc(cc1O)O)C	-7.4
model_263	Wighteone	CC(=CCc1c(O)cc2c(c1O)c(=O)c(co2)c1ccc(cc1)O)C	-7.4
model_397	Demethoxycapillarisin	Oc1ccc(cc1)Oc1cc(=O)c2c(o1)cc(cc2O)O	-7.4
model_426	Kazinol U	CC(=CCc1c(ccc(c1O)O)C1CCc2c(O1)cc(cc2)O)C	-7.4
model_446	6-Geranylnaringenin	CC(=CCc1c(O)cc2c(c1O)C(=O)CC(O2)c1ccc(cc1)O)CCC=C(C)C	-7.4
model_480	Pelargonidin chloride	Oc1ccc(cc1)c1[o+]c2cc(O)cc(c2cc1O)O.[Cl-]	-7.4
model_505	Neobavaisoflavone	CC(=CCc1cc(ccc1O)c1coc2c(c1=O)ccc(c2)O)C	-7.4
model_584	7,4'-Dihydroxyhomoisoflavanone	Oc1ccc(cc1)CC1COc2c(C1=O)ccc(c2)O	-7.4
model_62	Hispidulin	COc1c(O)cc2c(c1O)c(=O)cc(o2)c1ccc(cc1)O	-7.4
model_625	Warangalone	CC(=CCc1c2OC(C)(C)C=Cc2c(c2c1occ(c2=O)c1ccc(cc1)O)O)C	-7.4

model_641	Glepidotin B	CC(=CCc1c(O)cc(c2c1OC(c1ccccc1)C(C2=O)O)O)C	-7.4
model_645	Bidwillol A	COc1c(ccc(c1CC=C(C)C)O)C1=Cc2c(OC1)cc(cc2)O	-7.4
model_665	Auriculasin	CC(=CCc1c2OC(C)(C)C=Cc2c(c2c1occ(c2=O)c1ccc(c(c1)O)O)O)C	-7.4
model_683	6-Methyl-7-O-methylaromadendrin	COc1cc2OC(c3ccc(cc3)O)C(C(=O)c2c(c1C)O)O	-7.4
model_715	9-O-Methyl-4-hydroxyboeravinone B	COc1cc2oc3C(O)Oc4c(c3c(=O)c2c(c1C)O)cccc4O	-7.4
model_757	Glycyrrhisoflavone	CC(=CCc1cc(cc(c1O)O)c1coc2c(c1=O)c(O)cc(c2)O)C	-7.4
model_770	Boeravinone B	OC1Oc2ccccc2c2c1oc1cc(O)c(c(c1c2=O)O)C	-7.4
model_797	Ophiopogonanone E	COc1ccc(c(c1)O)CC1COc2c(C1=O)c(O)c(c(c2OC)O)C	-7.4
model_815	Boeravinone O	COc1cc(O)c2c(c1)oc1c(c2=O)c2cccc(c2OC1O)O	-7.4
model_981	Citrusinol	Oc1ccc(cc1)c1oc2c3C=CC(Oc3cc(c2c(=O)c1O)O)(C)C	-7.4
model_1003	Lupinalbin A	Oc1ccc2c(c1)oc1c2c(=O)c2c(o1)cc(cc2O)O	-7.3
model_1041	Karanjin	COc1c(oc2c(c1=O)ccc1c2cco1)c1ccccc1	-7.3
model_12	7-Hydroxy-3-(4-hydroxybenzylidene)chroman-4-one	Oc1ccc(cc1)C=C1COc2c(C1=O)ccc(c2)O	-7.3
model_183	Kumatakenin	COc1c(oc2c(c1=O)c(O)cc(c2)OC)c1ccc(cc1)O	-7.3
model_223	Dihydrorobinetin	Oc1ccc2c(c1)OC(C(C2=O)O)c1cc(O)c(c(c1)O)O	-7.3
model_224	Robtin	Oc1ccc2c(c1)OC(CC2=O)c1cc(O)c(c(c1)O)O	-7.3
model_313	Morusinol	Oc1ccc(c(c1)O)c1oc2c3C=CC(Oc3cc(c2c(=O)c1CCC(O)(C)C)O)(C)C	-7.3
model_356	Viscidulin I	Oc1cc(O)c2c(c1)oc(c(c2=O)O)c1c(O)cccc1O	-7.3
model_414	Eurycarpin A	CC(=CCc1c(O)ccc(c1O)c1coc2c(c1=O)ccc(c2)O)C	-7.3
model_442	Sappanone A	Oc1ccc2c(c1)OCC(=Cc1ccc(c(c1)O)O)C2=O	-7.3
model_554	Cyclocommunol	CC(=CC1Oc2cc(O)ccc2c2c1c(=O)c1c(o2)cc(cc1O)O)C	-7.3
model_577	3',5'-Diprenylgenistein	CC(=CCc1cc(cc(c1O)CC=C(C)C)c1coc2c(c1=O)c(O)cc(c2)O)C	-7.3
model_582	2H-1-Benzopyran-7-yloxy	Oc1ccc2c(c1)OC(CC2=O)c1ccccc1	-7.3
model_640	3,6-Dimethoxyapigenin	COc1c(O)cc2c(c1O)c(=O)c(c(o2)c1ccc(cc1)O)OC	-7.3
model_642	Sanggenon N	CC(=CCCC1(C)C=Cc2c(O1)ccc(c2O)C1CC(=O)c2c(O1)cc(cc2O)O)C	-7.3
model_728	Boeravinone E	Oc1ccc2c(c1)OC(c1c2c(=O)c2c(o1)cc(c(c2O)C)O)O	-7.3
model_837	(+/-)-Vestitol	COc1ccc(c(c1)O)C1COc2c(C1)ccc(c2)O	-7.3
model_848	Abyssinone V	CC(=CCc1cc(cc(c1O)CC=C(C)C)C1CC(=O)c2c(O1)cc(cc2O)O)C	-7.3
model_886	Licorisoflavan A	CC(=CCc1c(O)ccc(c1O)c1coc2c(c1=O)c(O)cc(c2)O)C	-7.3

model_941	Gancaonin G	COc1cc2occ(c(=O)c2c(c1CC=C(C)C)O)c1ccc(cc1)O	-7.3
model_989	Axillarin	COc1c(O)cc2c(c1O)c(=O)c(c(o2)c1ccc(c(c1)O)O)OC	-7.3
model_1037	Glabrol	CC(=CCc1cc(ccc1O)C1CC(=O)c2c(O1)c(CC=C(C)C)c(cc2)O)C	-7.2
model_1047	Eriosematin	CC(=CCc1c2OC(C)(C)C=Cc2c(c2c1occc2=O)O)C	-7.2
model_1079	Irilone	Oc1ccc(cc1)c1coc2c(c1=O)c(O)c1c(c2)OCO1	-7.2
model_11	Pinocembrin 7-acetate	CC(=O)Oc1cc2OC(CC(=O)c2c(c1)O)c1ccccc1	-7.2
model_112	Herbacetin	Oc1ccc(cc1)c1oc2c(O)c(O)cc(c2c(=O)c1O)O	-7.2
model_141	Erysubin B	OCC1(C)C=Cc2c(O1)cc1c(c2O)c(=O)c(co1)c1ccc(cc1)O	-7.2
model_156	5-Hydroxy-7-methoxy-3-(4-hydroxybenzylidene)chroman-4-one	COc1cc(O)c2c(c1)OCC(=Cc1ccc(cc1)O)C2=O	-7.2
model_220	Glabranin	CC(=CCc1c(O)cc(c2c1OC(CC2=O)c1ccccc1)O)C	-7.2
model_305	3,9-Dihydroxypterocarpan	Oc1ccc2c(c1)OCC1C2Oc2c1ccc(c2)O	-7.2
model_315	Erythrinin C	Oc1ccc(cc1)c1coc2c(c1=O)c(O)c1c(c2)OC(C1)C(O)(C)C	-7.2
model_333	Isomedicarpin	COc1ccc2c(c1)OC[C@@H]1[C@H]2Oc2c1cccc2	-7.2
model_336	Lupalbigenin	CC(=CCc1cc(ccc1O)c1coc2c(c1=O)c(O)c(c(c2)O)CC=C(C)C)C	-7.2
model_387	2'-Hydroxydaidzein	Oc1ccc(c(c1)O)c1coc2c(c1=O)ccc(c2)O	-7.2
model_427	Sulfuretin	Oc1ccc2c(c1)OC(=Cc1ccc(c(c1)O)O)C2=O	-7.2
model_428	Honyucitrin	CC(=CCc1cc(cc(c1O)CC=C(C)C)c1cc(=O)c2c(o1)cc(cc2O)O)C	-7.2
model_431	Barpisoflavone A	COc1cc(O)cc2c1c(=O)c(co2)c1ccc(cc1O)O	-7.2
model_575	Cathayanon H	CC(=CCc1cc(cc(c1O)CC=C(C)C)C1Oc2cc(O)cc(c2C(=O)C1O)O)C	-7.2
model_590	Neocyclomorusin	Oc1ccc2c(c1)OC(Cc1c2oc2c3C=CC(Oc3cc(c2c1=O)O)(C)C)C(O)(C)C	-7.2
model_6	3,7-O-Diacetylpinobanksin	CC(=O)Oc1cc(O)c2c(c1)OC(C(C2=O)OC(=O)C)c1ccccc1	-7.2
model_633	6,8-Diprenylgenistein	CC(=CCc1c(O)c(CC=C(C)C)c2c(c1O)c(=O)c(co2)c1ccc(cc1)O)C	-7.2
model_664	Syzalterin	Oc1ccc(cc1)c1cc(=O)c2c(o1)c(C)c(c(c2O)C)O	-7.2
model_838	Sativan	COc1cc(OC)ccc1C1COc2c(C1)ccc(c2)O	-7.2
model_1018	Odoriflavene	COc1c(OC)ccc(c1O)C1=Cc2c(OC1)cc(cc2)O	-7.1
model_1029	Glyasperin C	COc1c2CC(COc2cc(c1CC=C(C)C)O)c1ccc(cc1O)O	-7.1
model_104	3-Hydroxy-5,7-dimethoxy-3',4'-methylenedioxyflavan	COc1cc2OC(C(Cc2c(c1)OC)O)c1ccc2c(c1)OCO2	-7.1
model_1042	Dihydrolicoisoflavone	CC(=CCc1c(O)ccc(c1O)C1COc2c(C1=O)c(O)cc(c2)O)C	-7.1

model_1092	Glycinol	Oc1ccc2c(c1)OCC1(C2Oc2c1ccc(c2)O)O	-7.1
model_140	Erysubin A	Oc1ccc(cc1)c1coc2c(c1=O)c(O)c1c(c2)oc(c1)C(O)(C)C	-7.1
model_174	Sideroxylin	COc1c(C)c2oc(cc(=O)c2c(c1C)O)c1ccc(cc1)O	-7.1
model_21	2'-Hydroxygenistein	Oc1ccc(c(c1)O)c1coc2c(c1=O)c(O)cc(c2)O	-7.1
model_236	Pinostrobin	COc1cc2OC(CC(=O)c2c(c1)O)c1ccccc1	-7.1
model_238	Chrysin	Oc1cc(O)c2c(c1)oc(cc2=O)c1ccccc1	-7.1
model_249	Robinetin	Oc1ccc2c(c1)oc(c(c2=O)O)c1cc(O)c(c(c1)O)O	-7.1
model_269	Tectochrysin	COc1cc(O)c2c(c1)oc(cc2=O)c1ccccc1	-7.1
model_290	Galangin	Oc1cc(O)c2c(c1)oc(c(c2=O)O)c1ccccc1	-7.1
model_317	alpha-Isowighteone	Oc1ccc(cc1)c1coc2c(c1=O)c1OC(C)(C)CCc1c(c2)O	-7.1
model_318	Cirsimaritin	COc1cc2oc(cc(=O)c2c(c1OC)O)c1ccc(cc1)O	-7.1
model_322	5-Hydroxy-7-acetoxyflavone	CC(=O)Oc1cc(O)c2c(c1)oc(cc2=O)c1ccccc1	-7.1
model_331	2,3-Dehydrokievitone	CC(=CCc1c(O)cc(c2c1occ(c2=O)c1ccc(cc1O)O)O)C	-7.1
model_357	8-O-Demethyl-7-O-methyl-3,9-dihydropunctatin	COc1cc(O)c2c(c1O)OCC(C2=O)Cc1ccc(cc1)O	-7.1
model_370	5,7-Dihydroxy-2-isopropylchromone	Oc1cc(O)c2c(c1)oc(cc2=O)C(C)C	-7.1
model_377	Isothymusin	COc1c(O)c2oc(cc(=O)c2c(c1OC)O)c1ccc(cc1)O	-7.1
model_504	Norwogonin	Oc1cc(O)c2c(c1O)oc(cc2=O)c1ccccc1	-7.1
model_592	Eupatoletin	COc1cc2oc(c3ccc(c(c3)O)O)c(c(=O)c2c(c1OC)O)O	-7.1
model_595	7-Hydroxyflavone	Oc1ccc2c(c1)oc(cc2=O)c1ccccc1	-7.1
model_658	Pedalitin	COc1cc2oc(cc(=O)c2c(c1O)O)c1ccc(c(c1)O)O	-7.1
model_666	Isoerysenegalensein E	CC(=CCc1c(O)c(CC(C(=C)C)O)c(c2c1occ(c2=O)c1ccc(cc1)O)O)C	-7.1
model_716	1''-Hydroxyerythrinin C	Oc1ccc(cc1)c1coc2c(c1=O)c(O)c1c(c2)OC(C1O)C(O)(C)C	-7.1
model_734	Dihydrobaicalein	O=C1CC(Oc2c1c(O)c(c(c2)O)O)c1ccccc1	-7.1
model_775	8,3'-Diprenylapigenin	CC(=CCc1cc(ccc1O)c1cc(=O)c2c(o1)c(CC=C(C)C)c(cc2O)O)C	-7.1
model_827	Homoeriodictyol	COc1cc(ccc1O)C1CC(=O)c2c(O1)cc(cc2O)O	-7.1
model_849	7,8-Dihydroxyflavone	Oc1ccc2c(c1O)oc(cc2=O)c1ccccc1	-7.1
model_859	Isolupalbigenin	CC(=CCc1cc(ccc1O)c1coc2c(c1=O)c(O)cc(c2CC=C(C)C)O)C	-7.1
model_877	6-Methylgenistein	Oc1ccc(cc1)c1coc2c(c1=O)c(O)c(c(c2)O)C	-7.1
model_933	Dehydroglyasperin D	COc1c2C=C(COc2cc(c1CC=C(C)C)OC)c1ccc(cc1O)O	-7.1
model_942	Euchrestaflavanone B	CC(=CCc1cc(c(cc1O)O)C1CC(=O)c2c(O1)c(CC=C(C)C)c(cc2O)O)C	-7.1

model_990	Eriodictyol 7,3'-dimethyl ether	COc1cc2OC(CC(=O)c2c(c1)O)c1ccc(c(c1)OC)O	-7.1
model_1020	Pendulone	COC1=C(OC)C(=O)C=C(C1=O)C1COc2c(C1)ccc(c2)O	-7
model_1028	Glicoricone	COc1c(CC=C(C)C)c(O)cc(c1c1coc2c(c1=O)ccc(c2)O)O	-7
model_1067	Kushenol S	CC(=CCc1c(O)cc(c2c1OC(CC2=O)c1ccccc1O)O)C	-7
model_14	Pinocembrin diacetate	CC(=O)Oc1cc2OC(CC(=O)c2c(c1)OC(=O)C)c1ccccc1	-7
model_148	Amaronol B	COc1c(O)cc(cc1O)CC1(O)Oc2c(C1=O)c(O)cc(c2)O	-7
model_176	Isobavachin	CC(=CCc1c(O)ccc2c1OC(CC2=O)c1ccc(cc1)O)C	-7
model_192	4'-Demethyleucomin	Oc1ccc(cc1)C=C1COc2c(C1=O)c(O)cc(c2)O	-7
model_201	Oroxylin A	COc1c(O)cc2c(c1O)c(=O)cc(o2)c1ccccc1	-7
model_227	Brazilin/Brasilin	Oc1ccc2c(c1)OCC1(C2c2cc(O)c(cc2C1)O)O	-7
model_237	Pinocembrin	Oc1cc2OC(CC(=O)c2c(c1)O)c1ccccc1	-7
model_252	Baicalein	Oc1cc2oc(cc(=O)c2c(c1O)O)c1ccccc1	-7
model_254	Chrysoeriol	COc1cc(ccc1O)c1cc(=O)c2c(o1)cc(cc2O)O	-7
model_289	Pinobanksin	Oc1cc2OC(c3ccccc3)C(C(=O)c2c(c1)O)O	-7
model_335	5-Deoxycajanin	COc1ccc2c(c1)occ(c2=O)c1ccc(cc1O)O	-7
model_376	Leachianone A	COc1cc(O)ccc1C1CC(=O)c2c(O1)c(CC(C(=C)C)CC=C(C)C)<br>c(cc2O)O	-7
model_418	8-Prenyldaidzein	CC(=CCc1c(O)ccc2c1occ(c2=O)c1ccc(cc1)O)C	-7
model_443	8-Prenylnaringenin	CC(=CCc1c(O)cc(c2c1OC(CC2=O)c1ccc(cc1)O)O)C	-7
model_508	Corylifol C	CC(=CCc1c(O)ccc2c1oc(cc2=O)c1ccc(c(c1)O)O)C	-7
model_574	5,7-Dimethoxyflavanone	COc1cc2OC(CC(=O)c2c(c1)OC)c1ccccc1	-7
model_602	Izalpinine	COc1cc(O)c2c(c1)oc(c(c2=O)O)c1ccccc1	-7
model_613	Eriosemation	CC(=CCc1c(O)c(CC=C(C)C)c2c(c1O)c(=O)cco2)C	-7
model_614	4',7-Dihydroxyflavone	Oc1ccc2c(c1)oc(cc2=O)c1cc(c(c(c1)C(C)(C)C)O)C(C)(C)C	-7
model_627	Furowanin A	CC(=CCc1c2OC(Cc2c(c2c1occ(c2=O)c1ccc(c(c1)O)O)O)O)C(<br>O)(C)C)C	-7
model_630	Dalbergioidin	Oc1ccc(c(c1)O)C1COc2c(C1=O)c(O)cc(c2)O	-7
model_638	4'-O-Methyllicoflavanone	COc1ccc(cc1CC=C(C)C)C1CC(=O)c2c(O1)cc(cc2O)O	-7
model_646	5,7,4-Trihydroxy-3,6-dimethoxy-3-prenylflavone	COc1c(O)cc2c(c1O)c(=O)c(c(o2)c1ccc(c(c1)CC=C(C)C)O)O<br>C	-7
model_77	Chrysosplenol D	COc1c(oc2c(c1=O)c(O)c(c(c2)OC)OC)c1ccc(c(c1)O)O	-7
model_801	5,7-Dihydroxy-3-(4-hydroxy-3,5-dimethoxybenzyl)-6,8-dimethylchroman-4-one	COc1cc(CC2COc3c(C2=O)c(O)c(c(c3C)O)C)cc(c1O)OC	-7
model_825	Chrysindimethylether	COc1cc(OC)c2c(c1)oc(cc2=O)c1ccccc1	-7

model_828	Hematoxylin	Oc1cc2CC3(C(c2cc1O)c1ccc(c(c1OC3)O)O)O	-7
model_845	Cirsiliol	COc1cc2oc(cc(=O)c2c(c1OC)O)c1ccc(c(c1)O)O	-7
model_865	7,4'-Dihydroxy-8-methylflavan	Oc1ccc(cc1)C1CCc2c(O1)c(C)c(cc2)O	-7
model_983	Negletein	COc1cc2oc(cc(=O)c2c(c1O)O)c1ccccc1	-7
model_99	3',5,5',7-Tetrahydroxyflavanone	Oc1cc(O)cc(c1)C1CC(=O)c2c(O1)cc(cc2O)O	-7
model_151	5,7-Diacetoxy-8-methoxyflavone	COc1c(OC(=O)C)cc(c2c1oc(cc2=O)c1ccccc1)OC(=O)C	-6.9
model_179	Medicarpin	COc1ccc2c(c1)OC1C2COc2c1ccc(c2)O	-6.9
model_200	Alpinetin	COc1cc(O)cc2c1C(=O)CC(O2)c1ccccc1	-6.9
model_202	3'-O-Methylorobol	COc1cc(ccc1O)c1coc2c(c1=O)c(O)cc(c2)O	-6.9
model_226	Genistein	Oc1ccc(cc1)c1coc2c(c1=O)c(O)cc(c2)O	-6.9
model_234	Orobol	Oc1cc(O)c2c(c1)occ(c2=O)c1ccc(c(c1)O)O	-6.9
model_248	Daidzein	Oc1ccc(cc1)c1coc2c(c1=O)ccc(c2)O	-6.9
model_25	Blumeatin	COc1cc2OC(CC(=O)c2c(c1)O)c1cc(O)cc(c1)O	-6.9
model_271	Hesperetin	COc1ccc(cc1O)C1CC(=O)c2c(O1)cc(cc2O)O	-6.9
model_294	Penduletin	COc1c(oc2c(c1=O)c(O)c(c(c2)OC)OC)c1ccc(cc1)O	-6.9
model_321	5,7-Diacetoxyflavone	CC(=O)Oc1cc(OC(=O)C)c2c(c1)oc(cc2=O)c1ccccc1	-6.9
model_334	Artocarpin	COc1cc2oc(c3ccc(cc3O)O)c(c(=O)c2c(c1C=CC(C)C)O)CC=C(C)C	-6.9
model_34	5-Acetoxy-7-hydroxyflavone	CC(=O)Oc1cc(O)cc2c1c(=O)cc(o2)c1ccccc1	-6.9
model_372	Diosmetin	COc1ccc(cc1O)c1cc(=O)c2c(o1)cc(cc2O)O	-6.9
model_375	3,4'-Dihydroxy-3,5',7-trimethoxyflavan	COc1cc2OC(C(Cc2c(c1)OC)O)c1ccc(c(c1)OC)O	-6.9
model_406	Farrerol	Oc1ccc(cc1)C1CC(=O)c2c(O1)c(C)c(c(c2O)C)O	-6.9
model_434	Dracorhodin perchlorate	[O-][Cl](=O)(=O)=O.COc1c(C)c(O)cc2c1ccc([o+]2)c1ccccc1	-6.9
model_531	8-Prenylkaempferol	CC(=CCc1c(O)cc(c2c1oc(c1ccc(cc1)O)c(c2=O)O)O)C	-6.9
model_555	Onysilin	COc1cc2OC(CC(=O)c2c(c1OC)O)c1ccccc1	-6.9
model_557	Kazinol A	CC(=CCc1c(cc(c(c1O)O)CC=C(C)C)C1CCc2c(O1)cc(cc2)O)C	-6.9
model_571	Dihydrooroxylin A	COc1c(O)cc2c(c1O)C(=O)CC(O2)c1ccccc1	-6.9
model_580	Euchrestaflavanone A	CC(=CCc1cc(ccc1O)C1CC(=O)c2c(O1)c(CC=C(C)C)c(cc2O)O)C	-6.9
model_599	Cathayanon I	CC(=CCc1cc(c(c(c1O)CC=C(C)C)O)C1Oc2cc(O)cc(c2C(=O)C1O)O)C	-6.9
model_617	Homoferreirin	COc1ccc(c(c1)OC)C1COc2c(C1=O)c(O)cc(c2)O	-6.9
model_631	12-Deoxo-12alpha-acetoxyelliptone	COc1cc2c(cc1OC)OCC1C2C(OC(=O)C)c2c(O1)c1ccoc1cc2	-6.9

model_662	Angophorol	COc1c(C)c2OC(CC(=O)c2c(c1C)O)c1ccc(cc1)O	-6.9
model_667	Erysenegalensein E	CC(=CCc1c(O)c(CC(C(=C)C)O)c2c(c1O)c(=O)c(co2)c1ccc(cc1)O)C	-6.9
model_691	5,7,4'-Trihydroxy-8-methylflavanone	Oc1ccc(cc1)C1CC(=O)c2c(O1)c(C)c(cc2O)O	-6.9
model_710	Lupiwighteone	CC(=CCc1c(O)cc(c2c1occ(c2=O)c1ccc(cc1)O)O)C	-6.9
model_717	Mirabijalone D	COc1cc2oc3C(O)Oc4c(c3c(=O)c2c(c1C)O)ccc(c4)O	-6.9
model_824	Mosloflavone	COc1cc2oc(cc(=O)c2c(c1OC)O)c1ccccc1	-6.9
model_858	Maackiaflavanone	COc1cc(O)c2c(c1CC=C(C)C)OC(CC2=O)c1cc(CC=C(C)C)c(cc1O)O	-6.9
model_943	Dodovisone A	COc1c(O)cc2c(c1O)c(=O)c(c(o2)c1cc(CC=C(C)C)c2c(c1)CC(C(O2)(C)C)O)OC	-6.9
model_1010	Isoformononetin	COc1ccc2c(c1)occ(c2=O)c1ccc(cc1)O	-6.8
model_182	Cajanin	COc1cc(O)c2c(c1)occ(c2=O)c1ccc(cc1O)O	-6.8
model_240	Isosakuranetin	COc1ccc(cc1)C1CC(=O)c2c(O1)cc(cc2O)O	-6.8
model_295	4'-Hydroxywogonin	COc1c(O)cc(c2c1oc(cc2=O)c1ccc(cc1)O)O	-6.8
model_314	Wogonin	COc1c(O)cc(c2c1oc(cc2=O)c1ccccc1)O	-6.8
model_365	Prunetin	COc1cc(O)c2c(c1)occ(c2=O)c1ccc(cc1)O	-6.8
model_4	Noreugenin	Oc1cc(O)c2c(c1)oc(cc2=O)C	-6.8
model_452	Peonidin chloride	COc1cc(ccc1O)c1[o+]c2cc(O)cc(c2cc1O)O.[Cl-]	-6.8
model_454	Petunidin chloride	COc1cc(cc(c1O)O)c1[o+]c2cc(O)cc(c2cc1O)O.[Cl-]	-6.8
model_519	Licoflavone C	CC(=CCc1c(O)cc(c2c1oc(cc2=O)c1ccc(cc1)O)O)C	-6.8
model_547	7,3'-Dihydroxy-5'-methoxyisoflavone	COc1cc(O)cc(c1)c1coc2c(c1=O)ccc(c2)O	-6.8
model_601	Galangin 3-methyl ether	COc1c(oc2c(c1=O)c(O)cc(c2)O)c1ccccc1	-6.8
model_611	Sappanol	Oc1ccc2c(c1)OCC(C2O)(O)Cc1ccc(c(c1)O)O	-6.8
model_634	Lupinol C	CC(=CCc1c(O)cc2c(c1O)C(=O)C1(C(O2)Oc2c1ccc(c2)O)O)C	-6.8
model_655	Dodoviscin J	COc1cc(cc(c1O)CC=C(C)C)c1oc2cc(O)cc(c2c(=O)c1OC)O	-6.8
model_661	Phaseollidin hydrate	Oc1ccc2c(c1)OCC1C2Oc2c1ccc(c2CCC(O)(C)C)O	-6.8
model_723	Sophoflavescenol	COc1cc(O)c(c2c1c(=O)c(O)c(o2)c1ccc(cc1)O)CC=C(C)C	-6.8
model_724	Isoxanthohumol	COc1cc(O)c(c2c1C(=O)CC(O2)c1ccc(cc1)O)CC=C(C)C	-6.8
model_727	3'-Methoxydaidzein	COc1cc(ccc1O)c1coc2c(c1=O)ccc(c2)O	-6.8
model_733	6,7,4'-Trihydroxyisoflavone	Oc1ccc(cc1)c1coc2c(c1=O)cc(c(c2)O)O	-6.8
model_752	Isoanhydroicaritin	COc1cc(O)c2c(c1CC=C(C)C)oc(c(c2=O)O)c1ccc(cc1)O	-6.8
model_823	Dehydroglyasperin C	COc1c2C=C(COc2cc(c1CC=C(C)C)O)c1ccc(cc1O)O	-6.8

model_841	Licoricone	COc1c(CC=C(C)C)c(OC)cc(c1c1coc2c(c1=O)ccc(c2)O)O	-6.8
model_860	Glyasperin D	COc1c2CC(COc2cc(c1CC=C(C)C)OC)c1ccc(cc1O)O	-6.8
model_93	Biochanin A	COc1ccc(cc1)c1coc2c(c1=O)c(O)cc(c2)O	-6.8
model_1093	Norwogenin 5,7,8-trimethyl ether	COc1cc(OC)c2c(c1OC)oc(cc2=O)c1ccccc1	-6.7
model_119	Tectorigenin	COc1c(O)cc2c(c1O)c(=O)c(co2)c1ccc(cc1)O	-6.7
model_155	Velutin	COc1cc(O)c2c(c1)oc(cc2=O)c1ccc(c(c1)OC)O	-6.7
model_198	Moslosooflavone	COc1c(OC)cc(c2c1oc(cc2=O)c1ccccc1)O	-6.7
model_205	Naringenin triacetate	CC(=O)Oc1ccc(cc1)C1CC(=O)c2c(O1)cc(cc2OC(=O)C)OC(=O)C	-6.7
model_217	Skullcapflavone I	COc1cc(O)c2c(c1OC)oc(cc2=O)c1ccccc1O	-6.7
model_232	Isorhamnetin	COc1cc(ccc1O)c1oc2cc(O)cc(c2c(=O)c1O)O	-6.7
model_241	Acacetin	COc1ccc(cc1)c1cc(=O)c2c(o1)cc(cc2O)O	-6.7
model_301	Isosativan	COc1ccc2c(c1)OCC(C2)c1ccc(cc1O)OC	-6.7
model_303	Tamarixetin	COc1ccc(cc1O)c1oc2cc(O)cc(c2c(=O)c1O)O	-6.7
model_329	Dihydrotamarixetin	COc1ccc(cc1O)C1Oc2cc(O)cc(c2C(=O)C1O)O	-6.7
model_33	Glycitein	COc1cc2c(cc1O)occ(c2=O)c1ccc(cc1)O	-6.7
model_330	Sepinol	COc1c(O)cc(cc1O)C1Oc2cc(O)ccc2C(=O)C1O	-6.7
model_341	2',5,6',7-Tetrahydroxyflavanone	Oc1cc2OC(CC(=O)c2c(c1)O)c1c(O)cccc1O	-6.7
model_415	7,3'-Dihydroxy-4'-methoxyflavan	COc1ccc(cc1O)C1CCc2c(O1)cc(cc2)O	-6.7
model_503	Rivularin	COc1cccc(c1c1cc(=O)c2c(o1)c(OC)c(cc2O)OC)O	-6.7
model_522	Kuwanon E	CC(=CCc1cc(c(cc1O)O)C1CC(=O)c2c(O1)cc(cc2O)O)CCC=C(C)C	-6.7
model_529	Kushenol L	CC(=CCc1c2OC(c3ccc(cc3O)O)C(C(=O)c2c(c(c1O)CC=C(C)C)O)O)C	-6.7
model_551	(+/-)-Sigmoidin A	CC(=CCc1c(cc(c(c1O)O)CC=C(C)C)C1CC(=O)c2c(O1)cc(cc2O)O)C	-6.7
model_564	Kurarinol	COc1cc(O)c(c2c1C(=O)CC(O2)c1ccc(cc1O)O)CC(C(=C)C)CCC(O)(C)C	-6.7
model_620	Isochandalone	CC(=CCc1c(O)cc2c(c1O)c(=O)c(co2)c1ccc2c(c1)C=CC(O2)(C)C)C	-6.7
model_650	Dodoviscin	OCC(CCc1cc(ccc1O)c1oc2cc(O)cc(c2c(=O)c1OC)O)C	-6.7
model_686	Dihydrodaidzein	Oc1ccc(cc1)C1COc2c(C1=O)ccc(c2)O	-6.7
model_754	Pratensein	COc1ccc(cc1O)c1coc2c(c1=O)c(O)cc(c2)O	-6.7
model_790	6-Hydroxywogonin	COc1c(O)c(O)c(c2c1oc(cc2=O)c1ccccc1)O	-6.7
model_802	3-(2,4-Dihydroxybenzyl)-5-hydroxy-7,8-dimethoxy-6-methylchroman-4-one	COc1c2OCC(C(=O)c2c(c(c1OC)C)O)Cc1ccc(cc1O)O	-6.7

model_822	Trilepisflavan	COc1cc(ccc1OC)C1CCc2c(O1)cc(cc2)O	-6.7
model_972	Montixanthone	COc1cc(O)cc2c1c(=O)c1c(o2)cc(c(c1)O)O	-6.7
model_1034	5-Methyl-7-methoxyisoflavone	COc1cc(C)c2c(c1)occ(c2=O)c1ccccc1	-6.6
model_1064	Parvisoflavanone	COc1c(ccc(c1OC)O)C1COc2c(C1=O)c(O)cc(c2)O	-6.6
model_1070	5,7,2',4'-Tetrahydroxy-8,3'-di(gamma,gamma-dimethylallyl)-isoflavanone	CC(=CCc1c(O)ccc(c1O)C1COc2c(C1=O)c(O)cc(c2CC=C(C)C)O)C	-6.6
model_135	Formononetin	COc1ccc(cc1)c1coc2c(c1=O)ccc(c2)O	-6.6
model_177	6-Methoxywogonin	COc1c(O)c(OC)c2c(c1O)c(=O)cc(o2)c1ccccc1	-6.6
model_185	Pachypodol	COc1cc(O)c2c(c1)oc(c(c2=O)OC)c1ccc(c(c1)OC)O	-6.6
model_20	5-Hydroxy-7,8-dimethoxyflavanone	COc1c(OC)cc(c2c1OC(CC2=O)c1ccccc1)O	-6.6
model_207	Phaseollidin	CC(=CCc1c(O)ccc2c1OC1C2COc2c1ccc(c2)O)C	-6.6
model_270	Tricin	COc1cc(cc(c1O)OC)c1cc(=O)c2c(o1)cc(cc2O)O	-6.6
model_274	3-O-Acetylpinobanksin	CC(=O)OC1C(Oc2c(C1=O)c(O)cc(c2)O)c1ccccc1	-6.6
model_325	Trifolirhizin	OCC1OC(Oc2ccc3c(c2)OCC2C3Oc3c2cc2c(c3)OCO2)C(C(C1O)O)O	-6.6
model_339	2',3,5,6',7-Pentahydroxyflavanone	Oc1cc2OC(c3c(O)cccc3O)C(C(=O)c2c(c1)O)O	-6.6
model_416	Jaceosidin	COc1cc(ccc1O)c1cc(=O)c2c(o1)cc(c(c2O)OC)O	-6.6
model_500	Viscidulin II	COc1cc(O)c2c(c1OC)oc(cc2=O)c1c(O)cccc1O	-6.6
model_528	Leachianone G	CC(=CCc1c(O)cc(c2c1OC(CC2=O)c1ccc(cc1O)O)O)C	-6.6
model_546	Kushenol E	CC(=CCc1c2OC(CC(=O)c2c(c(c1O)CC=C(C)C)O)c1ccc(cc1O)O)C	-6.6
model_597	3'-Deoxy-4-O-methylsappanol	COC1c2ccc(cc2OCC1(O)Cc1ccc(cc1)O)O	-6.6
model_659	Derrisisoflavone B	CC(=CCc1c(O)cc2c(c1O)c(=O)c(co2)c1ccc(c(c1)CC(C(=C)C)O)O)C	-6.6
model_8	4-O-Methylsappanol	COC1c2ccc(cc2OCC1(O)Cc1ccc(c(c1)O)O)O	-6.6
model_831	5,7-Dihydroxy-3',4',5'-trimethoxyflavone	COc1c(OC)cc(cc1OC)c1cc(=O)c2c(o1)cc(cc2O)O	-6.6
model_136	Ermanin	COc1ccc(cc1)c1oc2cc(O)cc(c2c(=O)c1OC)O	-6.5
model_195	Anhydroicaritin	COc1ccc(cc1)c1oc2c3CCC(Oc3cc(c2c(=O)c1O)O)(C)C	-6.5
model_197	Noricaritin	Oc1ccc(cc1)c1oc2c(CCC(O)(C)C)c(O)cc(c2c(=O)c1O)O	-6.5
model_209	Naringenintrimethyl ether	COc1ccc(cc1)C1CC(=O)c2c(O1)cc(cc2OC)OC	-6.5
model_251	Kaempferide	COc1ccc(cc1)c1oc2cc(O)cc(c2c(=O)c1O)O	-6.5
model_368	5-Hydroxy-7-acetoxy-8-methoxyflavone	COc1c(OC(=O)C)cc(c2c1oc(cc2=O)c1ccccc1)O	-6.5
model_401	Isopedicin	COc1c2OC(CC(=O)c2c(c(c1OC)O)OC)c1ccccc1	-6.5

model_438	Kurarinone	COc1cc(O)c(c2c1C(=O)CC(O2)c1ccc(cc1O)O)CC(C(=C)C)CC=C(C)C	-6.5
model_440	Sophoraflavanone G	CC(=CCC(C(=C)C)Cc1c(O)cc(c2c1OC(CC2=O)c1ccc(cc1O)O)O)C	-6.5
model_445	6,8-Diprenylnaringenin	CC(=CCc1c2OC(CC(=O)c2c(c(c1O)CC=C(C)C)O)c1ccc(cc1)O)C	-6.5
model_45	Calycosin	COc1ccc(cc1O)c1coc2c(c1=O)ccc(c2)O	-6.5
model_453	Malvidin chloride	COc1cc(cc(c1O)OC)c1[o+]c2cc(O)cc(c2cc1O)O.[Cl-]	-6.5
model_502	Skullcapflavone II	COc1cccc(c1c1cc(=O)c2c(o1)c(OC)c(c(c2O)OC)OC)O	-6.5
model_558	Isothymonin	COc1cc(ccc1O)c1cc(=O)c2c(o1)c(O)c(c(c2O)OC)OC	-6.5
model_649	Aliarin	COc1ccc(cc1CCC(O)C)c1oc2cc(O)c(c(c2c(=O)c1OC)O)OC	-6.5
model_813	Sideritoflavone	COc1c(OC)c(OC)c(c2c1oc(cc2=O)c1ccc(c(c1)O)O)O	-6.5
model_820	Hesperetin-7-methyl ether	COc1cc2OC(CC(=O)c2c(c1)O)c1ccc(c(c1)O)OC	-6.5
model_868	2',4'-Dihydroxy-7-methoxy-8-prenylflavan	COc1ccc2c(c1CC=C(C)C)OC(CC2)c1ccc(cc1O)O	-6.5
model_920	Pilloin	COc1cc(O)c2c(c1)oc(cc2=O)c1ccc(c(c1)O)OC	-6.5
model_979	Trimethylapigenin	COc1ccc(cc1)c1cc(=O)c2c(o1)cc(cc2OC)OC	-6.5
model_1	Pectolinarigenin	COc1ccc(cc1)c1cc(=O)c2c(o1)cc(c(c2O)OC)O	-6.4
model_1030	Gancaonin N	COc1ccc(c(c1)O)c1coc2c(c1=O)c(O)c(c(c2)O)CC=C(C)C	-6.4
model_16	2',5,7-Trihydroxy-8-methoxyflavanone	COc1c(O)cc(c2c1OC(CC2=O)c1ccccc1O)O	-6.4
model_175	Eucalyptin	COc1ccc(cc1)c1cc(=O)c2c(o1)c(C)c(c(c2O)C)OC	-6.4
model_184	Quercetin 3,4'-dimethyl ether	COc1ccc(cc1O)c1oc2cc(O)cc(c2c(=O)c1OC)O	-6.4
model_2	7-Hydroxy-2',5,8-trimethoxyflavanone	COc1ccccc1C1CC(=O)c2c(O1)c(OC)c(cc2OC)O	-6.4
model_22	3',4',7-Trimethoxyflavan	COc1cc(cc(c1OC)O)C1CCc2c(O1)c(O)c(cc2)OC	-6.4
model_281	Ombuin	COc1cc(O)c2c(c1)oc(c(c2=O)O)c1ccc(c(c1)O)OC	-6.4
model_288	7-Hydroxy-5,8-dimethoxyflavanone	COc1c(O)cc(c2c1OC(CC2=O)c1ccccc1)OC	-6.4
model_344	Visnagin	COc1c2c(=O)cc(oc2cc2c1cco2)C	-6.4
model_424	5,7,3'-Trihydroxy-4'-methoxy-8-prenylflavanone	COc1ccc(cc1O)C1CC(=O)c2c(O1)c(CC=C(C)C)c(cc2O)O	-6.4
model_435	Kushenol A	CC(=CCC(C(=C)C)Cc1c(O)cc(c2c1OC(CC2=O)c1ccccc1O)O)C	-6.4
model_436	Kushenol I	COc1cc(O)c(c2c1C(=O)C(O)C(O2)c1ccc(cc1O)O)CC(C(=C)C)CC=C(C)C	-6.4
model_5	Jaceidin	COc1cc(ccc1O)c1oc2cc(O)c(c(c2c(=O)c1OC)O)OC	-6.4
model_570	4'-Hydroxy-7-methoxyflavan	COc1ccc2c(c1)OC(CC2)c1ccc(cc1)O	-6.4
model_616	Gancaonin M	COc1ccc(cc1)c1coc2c(c1=O)c(O)cc(c2CC=C(C)C)O	-6.4

model_708	2',7-Dihydroxy-5,8-dimethoxyflavanone	COc1c(O)cc(c2c1OC(CC2=O)c1ccccc1O)OC	-6.4
model_713	Ayanin	COc1cc(O)c2c(c1)oc(c(c2=O)OC)c1ccc(c(c1)O)OC	-6.4
model_743	6-Demethoxytangeretin	COc1ccc(cc1)c1cc(=O)c2c(o1)c(OC)c(cc2OC)OC	-6.4
model_772	Toxicarolisoflavone	COc1cc(OC)c(cc1c1coc2c(c1=O)c(O)cc1c2C=CC(O1)(C)C)OC	-6.4
model_809	Ladanein	COc1ccc(cc1)c1cc(=O)c2c(o1)cc(c(c2O)O)OC	-6.4
model_811	Licoricidin	COc1c2CC(COc2cc(c1CC=C(C)C)O)c1ccc(c(c1O)CC=C(C)C)O	-6.4
model_89	3,5-Dihydroxy-4',7-dimethoxyflavone	COc1ccc(cc1)c1oc2cc(OC)cc(c2c(=O)c1O)O	-6.4
model_934	7-O-Methyleucomol	COc1ccc(cc1)CC1(O)COc2c(C1=O)c(O)cc(c2)OC	-6.4
model_1053	Daidzein diacetate	CC(=O)Oc1ccc(cc1)c1coc2c(c1=O)ccc(c2)OC(=O)C	-6.3
model_149	Desmethoxycentaureidin	COc1ccc(cc1O)c1cc(=O)c2c(o1)cc(c(c2O)OC)O	-6.3
model_166	4',7-Di-O-methylnaringenin	COc1ccc(cc1)C1CC(=O)c2c(O1)cc(cc2O)OC	-6.3
model_173	5,7,3'-Trihydroxy-6,4',5'-trimethoxyflavanone	COc1c(O)cc(cc1OC)C1CC(=O)c2c(O1)cc(c(c2O)OC)O	-6.3
model_208	Cimifugin	COc1c2CC(Oc2cc2c1c(=O)cc(o2)CO)C(O)(C)C	-6.3
model_265	7,4'-Di-O-methylapigenin / Apigenin 7,4'-dimethyl ether	COc1ccc(cc1)c1cc(=O)c2c(o1)cc(cc2O)OC	-6.3
model_273	2-Hydroxy-7-O-methylscillascillin	COc1cc(O)c2c(c1)OC(C1(C2=O)Cc2c1cc1c(c2)OCO1)O	-6.3
model_302	Chrysosplenetin	COc1cc2oc(c3ccc(c(c3)OC)O)c(c(=O)c2c(c1OC)O)OC	-6.3
model_354	8-Methoxybonducellin	COc1ccc(cc1)C=C1COc2c(C1=O)ccc(c2OC)O	-6.3
model_355	Viscidulin III/ Ganhuangenin	COc1c(O)ccc(c1c1cc(=O)c2c(o1)c(OC)c(cc2O)O)O	-6.3
model_405	5,7-Dihydroxychromone	Oc1cc(O)c2c(c1)occc2=O	-6.3
model_420	4-Hydroxycoumarin	O=c1cc(O)c2c(o1)cccc2	-6.3
model_421	Centaureidin	COc1ccc(cc1O)c1oc2cc(O)c(c(c2c(=O)c1OC)O)OC	-6.3
model_501	5,2',5'-Trihydroxy-6,7,8-trimethoxyflavone	COc1c(OC)c(OC)c(c2c1oc(cc2=O)c1cc(O)ccc1O)O	-6.3
model_521	Tephrosin	COc1cc2c(cc1OC)OCC1C2(O)C(=O)c2c(O1)c1C=CC(Oc1cc2)(C)C	-6.3
model_527	Kushenol W	COc1cc(C2CC(=O)c3c(O2)c(CC=C(C)C)c(cc3O)O)c(cc1O)O	-6.3
model_541	8-Methoxykaempferol / Sexangularetin	COc1c(O)cc(c2c1oc(c1ccc(cc1)O)c(c2=O)O)O	-6.3
model_705	Tsugafolin	COc1ccc(cc1)C1CC(=O)c2c(O1)cc(cc2OC)O	-6.3
model_729	Kaempferol 3,7,4'-trimethylether	COc1ccc(cc1)c1oc2cc(OC)cc(c2c(=O)c1OC)O	-6.3
model_745	5,7,3'-Trihydroxy-6,4',5'-trimethoxyflavone	COc1c(O)cc(cc1OC)c1cc(=O)c2c(o1)cc(c(c2O)OC)O	-6.3
model_771	Alpha-Toxicarol	COc1cc2OCC3C(c2cc1OC)C(=O)c1c(O3)c2C=CC(Oc2cc1O)(C)C	-6.3

model_117	Naringenin-4',7-diacetate	CC(=O)Oc1ccc(cc1)C1CC(=O)c2c(O1)cc(cc2O)OC(=O)C	-6.2
model_165	5-Hydroxy-3',4',7-trimethoxyflavone	COc1cc(O)c2c(c1)oc(cc2=O)c1ccc(c(c1)OC)OC	-6.2
model_225	Norkhellol	OCc1cc(=O)c2c(o1)cc1c(c2O)cco1	-6.2
model_255	Angelicain	OCc1cc(=O)c2c(o1)cc1c(c2O)CC(O1)C(O)(C)C	-6.2
model_28	3',5,5',7-Tetrahydroxy-4',6-dimethoxyflavone	COc1c(O)cc(cc1O)c1cc(=O)c2c(o1)cc(c(c2O)OC)O	-6.2
model_293	8-Demethyleucalyptin	COc1ccc(cc1)c1cc(=O)c2c(o1)cc(c(c2O)C)OC	-6.2
model_337	3-Hydroxy-4',5,7-trimethoxyflavanone	COc1ccc(cc1)C1Oc2cc(OC)cc(c2C(=O)C1O)OC	-6.2
model_338	Blumeatin B	COc1cc2OC(c3ccc(c(c3)O)OC)C(C(=O)c2c(c1)O)O	-6.2
model_366	Eupatilin	COc1cc(ccc1OC)c1cc(=O)c2c(o1)cc(c(c2O)OC)O	-6.2
model_404	Capillarisin	COc1c(O)cc2c(c1O)c(=O)cc(o2)Oc1ccc(cc1)O	-6.2
model_439	2'-Methoxykurarinone	COc1cc(O)c(c2c1C(=O)CC(O2)c1ccc(cc1OC)O)CC(C(=C)C)CC=C(C)C	-6.2
model_755	Isosinensetin	COc1cc(ccc1OC)c1cc(=O)c2c(o1)c(OC)c(cc2OC)OC	-6.2
model_980	Tetramethylkaempferol	COc1ccc(cc1)c1oc2cc(OC)cc(c2c(=O)c1OC)OC	-6.2
model_1031	4''-methyloxy-Daidzin	OCC1OC(Oc2ccc3c(c2)occ(c3=O)c2ccc(cc2)O)C(C(C1OC)O)O	-6.1
model_398	Homopterocarpin	COc1ccc2c(c1)OCC1C2Oc2c1ccc(c2)OC	-6.1
model_419	Cirsilineol	COc1cc2oc(cc(=O)c2c(c1OC)O)c1ccc(c(c1)OC)O	-6.1
model_532	Afrormosine	COc1ccc(cc1)c1coc2c(c1=O)cc(c(c2)O)OC	-6.1
model_573	Sophoraflavanone C	CC(=CCc1c(O)cc(c2c1OC(CC2=O)c1ccc(cc1O)O)O)CCC=C(C)C	-6.1
model_632	Deguelin	COc1cc2c(cc1OC)OCC1C2C(=O)c2c(O1)c1C=CC(Oc1cc2)(C)C	-6.1
model_657	5,7,4'-Tri-O-methylcatechin	COc1cc2OC(c3ccc(c(c3)O)OC)C(Cc2c(c1)OC)O	-6.1
model_880	Pierreione B	COc1cc(ccc1OCC(C(O)(C)C)O)c1coc2c(c1=O)cc1c(c2)OC(C=C1)(C)C	-6.1
model_935	7,3'-Di-O-methylorobol	COc1cc(O)c2c(c1)occ(c2=O)c1ccc(c(c1)OC)O	-6.1
model_1017	Colutehydroquinone	COc1ccc2c(c1)OCC(C2)c1cc(O)c(c(c1O)OC)OC	-6
model_1024	4',5,6,7-Tetramethoxyflavone	COc1ccc(cc1)c1cc(=O)c2c(o1)cc(c(c2OC)OC)OC	-6
model_1045	Irisolidone	COc1ccc(cc1)c1coc2c(c1=O)c(O)c(c(c2)O)OC	-6
model_1085	Hamaudol	Cc1cc(=O)c2c(o1)cc1c(c2O)CC(C(O1)(C)C)O	-6
model_15	3',5-Dihydroxy-4',5',6,7-tetramethoxyflavone	COc1c(O)cc(cc1OC)c1cc(=O)c2c(o1)cc(c(c2O)OC)OC	-6
model_190	Prudomestin	COc1ccc(cc1)c1oc2c(OC)c(O)cc(c2c(=O)c1O)O	-6
model_196	Icaritin	COc1ccc(cc1)c1oc2c(CC=C(C)C)c(O)cc(c2c(=O)c1O)O	-6

model_342	2',5,6',7-Tetraacetoxyflavanone	CC(=O)Oc1cc(OC(=O)C)c2c(c1)OC(CC2=O)c1c(cccc1OC(=O)C)OC(=O)C	-6
model_345	4',5-Dihydroxy-3',5',6,7-tetramethoxyflavone	COc1cc2oc(cc(=O)c2c(c1OC)O)c1cc(OC)c(c(c1)OC)O	-6
model_348	Eupatorin	COc1cc2oc(cc(=O)c2c(c1OC)O)c1ccc(c(c1)O)OC	-6
model_383	Isolicoflavonol	CC(=CCc1cc(ccc1O)c1oc2cc(O)cc(c2c(=O)c1O)O)C	-6
model_50	Eucalyptin acetate	COc1c(C)c2oc(cc(=O)c2c(c1C)OC(=O)C)c1ccc(cc1)OC	-6
model_507	Corylifol A	CC(=CCc1cc(ccc1O)c1coc2c(c1=O)ccc(c2)O)CCC=C(C)C	-6
model_544	Limocitrin	COc1cc(ccc1O)c1oc2c(OC)c(O)cc(c2c(=O)c1O)O	-6
model_622	Dehydrodeguelin	COc1cc2c(cc1OC)OCc1c2c(=O)c2c(o1)c1C=CC(Oc1cc2)(C)C	-6
model_1013	7-O-Methylbiochanin A	COc1ccc(cc1)c1coc2c(c1=O)c(O)cc(c2)OC	-5.9
model_123	Epimedin A	COc1ccc(cc1)c1cc(=O)c2c(o1)cc(c(c2O)OC)OC	-5.9
model_328	6-Acetyl-2,2-dimethylchroman-4-one	O=C1CC(C)(C)Oc2c1cc(cc2)C(=O)C	-5.9
model_736	Irigenin	COc1cc(cc(c1OC)O)c1coc2c(c1=O)c(O)c(c(c2)O)OC	-5.9
model_79	Sinensetin	COc1ccc(cc1OC)c1cc(=O)c2c(o1)cc(c(c2OC)OC)OC	-5.9
model_1051	3'-Demethylnobiletin	COc1ccc(cc1O)c1cc(=O)c2c(o1)c(OC)c(c(c2OC)OC)OC	-5.8
model_137	2',4',5'-Trimethoxy-2",2"-dimethylpyrano[5",6":6,7]isoflavone	COc1cc(OC)c(cc1c1coc2c(c1=O)cc1c(c2)OC(C=C1)(C)C)OC	-5.8
model_647	11-Hydroxytephrosin	COc1cc2OCC3C(c2cc1OC)(O)C(=O)c1c(O3)c2C=CC(Oc2cc1O)(C)C	-5.8
model_740	Gardenin B	COc1ccc(cc1)c1cc(=O)c2c(o1)c(OC)c(c(c2O)OC)OC	-5.8
model_836	Alpinumisoflavone acetate	CC(=O)Oc1ccc(cc1)c1coc2c(c1=O)c(O)c1c(c2)OC(C=C1)(C)C	-5.8
model_899	Daidzein dimethyl ether	COc1ccc(cc1)c1coc2c(c1=O)ccc(c2)OC	-5.8
model_915	Corymbosin	COc1cc(O)c2c(c1)oc(cc2=O)c1cc(OC)c(c(c1)OC)OC	-5.8
model_94	5,7-Dihydroxy-3,4',8-trimethoxyflavone	COc1ccc(cc1)c1oc2c(OC)c(O)cc(c2c(=O)c1OC)O	-5.8
model_114	Irisflorentin	COc1cc(cc(c1OC)OC)c1coc2c(c1=O)c(OC)c1c(c2)OCO1	-5.7
model_264	5,7-Diacetoxy-3,4',8-trimethoxyflavone	COc1ccc(cc1)c1oc2c(OC)c(OC(=O)C)cc(c2c(=O)c1OC)OC(=O)C	-5.7
model_389	Tangeretin	COc1ccc(cc1)c1cc(=O)c2c(o1)c(OC)c(c(c2OC)OC)OC	-5.7
model_41	Ononin	OCC1OC(Oc2ccc3c(c2)occ(c3=O)c2ccc(cc2)OC)C(C(C1O)O)O	-5.7
model_55	Kaempferol 3,4,7-triacetate	CC(=O)Oc1ccc(cc1)c1oc2cc(OC(=O)C)cc(c2c(=O)c1OC(=O)C)O	-5.7
model_583	2H-1-Benzopyran-5-ol	CCCCCc1ccc2c(c1O)C=CC(O2)(C)C	-5.7

model_669	Dehydrotoxicarol	COc1cc2OCc3c(c2cc1OC)c(=O)c1c(o3)c2C=CC(Oc2cc1O)(C)C	-5.7
model_800	Ophiopogonanone F	COc1ccc(c(c1)O)CC1COc2c(C1=O)c(OC)c(c(c2OC)O)C	-5.7
model_847	Arteanoflavone	COc1c(OC)cc(cc1OC)c1cc(=O)c2c(o1)cc(c(c2O)OC)O	-5.7
model_910	Nevadensin	COc1ccc(cc1)c1cc(=O)c2c(o1)c(OC)c(c(c2O)OC)O	-5.7
model_1050	Hexamethylquercetagetin	COc1ccc(cc1OC)c1oc2cc(OC)c(c(c2c(=O)c1OC)OC)OC	-5.6
model_164	Vitexicarpin/Casticin	COc1cc2oc(c3ccc(c(c3)O)OC)c(c(=O)c2c(c1OC)O)OC	-5.6
model_229	Artemetin	COc1ccc(cc1OC)c1oc2cc(OC)c(c(c2c(=O)c1OC)O)OC	-5.6
model_751	5-O-Demethylnobiletin	COc1cc(ccc1OC)c1cc(=O)c2c(o1)c(OC)c(c(c2O)OC)OC	-5.6
model_189	Isoapetalic acid	CCCC(c1c2OC(C)C(C(=O)c2c(c2c1OC(C)(C)C=C2)O)C)CC(=O)O	-5.5
model_364	Artemetin acetate	COc1ccc(cc1OC)c1oc2cc(OC)c(c(c2c(=O)c1OC)OC(=O)C)OC	-5.5
model_131	8-Hydroxy-3,5,7,3',4',5'-hexamethoxyflavone	COc1cc(cc(c1OC)OC)c1oc2c(O)c(OC)cc(c2c(=O)c1OC)OC	-5.4
model_343	Khellin	COc1c2c(=O)cc(oc2c(c2c1cco2)OC)C	-5.4
model_968	5'-Methoxynobiletin	COc1c(OC)cc(cc1OC)c1cc(=O)c2c(o1)c(OC)c(c(c2OC)OC)OC	-5.4
model_256	Araneosol	COc1ccc(cc1)c1oc2c(OC)c(O)c(c(c2c(=O)c1OC)O)OC	-5.3
model_1009	3,3',4',5,6,7,8-heptamethoxyflavone	COc1cc(ccc1OC)c1oc2c(OC)c(OC)c(c(c2c(=O)c1OC)OC)OC	-5.2
model_636	Maltol	O=c1ccoc(c1O)C	-5.2
model_109	3',4',5',3,5,6,7-Heptamethoxyflavone	COc1c(OC)cc(cc1OC)c1oc2cc(OC)c(c(c2c(=O)c1OC)OC)OC	-5
model_36	Exoticin	COc1c(OC)cc(cc1OC)c1oc2c(OC)c(OC)c(c(c2c(=O)c1OC)OC)OC	-5

Supplementary Table S2: Kinase targets of top 4 lead flavonoids (The top 4 lead flavonoids, 5-Dehydroxyparatocarpin K, Carpachromene, Sanggenone H, and Kuwanol C were represented as L1, L2, L3, and L4 respectively).

Target	Common name	Uniprot ID	ChEMBL ID	L1	L2	L3	L4
3-phosphoinositide dependent protein kinase-1	PDPK1	O15530	CHEMBL2534			Yes	
CaM kinase II	CAMK2D	Q13557	CHEMBL2801				Yes
Casein kinase I gamma 1	CSNK1G1	Q9HCP0	CHEMBL2426			Yes	
Casein kinase II alpha	CSNK2A1	P68400	CHEMBL3629		Yes		
CDC7/DBF4 (Cell division cycle 7-related protein kinase/Activator of S phase kinase)	CDC7	O00311	CHEMBL5443	Yes			
CDK2/Cyclin A	CCNA2 CDK2	P20248 P24941	CHEMBL3038469				Yes
c-Jun N-terminal kinase 3	MAPK10	P53779	CHEMBL2637				Yes
Cyclin-dependent kinase 1	CDK1	P06493	CHEMBL308	Yes			
Cyclin-dependent kinase 2	CDK2	P24941	CHEMBL301			Yes	
Cyclin-dependent kinase 2/cyclin E1	CCNE1 CDK2	P24864 P24941	CHEMBL1907605				Yes
Cyclin-dependent kinase 4	CDK4	P11802	CHEMBL331			Yes	
Cyclin-dependent kinase 5/CDK5 activator 1	CDK5R1 CDK5	Q15078 Q00535	CHEMBL1907600		Yes		Yes
Cyclin-dependent kinase 6	CDK6	Q00534	CHEMBL2508		Yes		
Dual specificity protein kinase TTK	TTK	P33981	CHEMBL3983				Yes
Ephrin receptor	EPHB4	P54760	CHEMBL5147			Yes	
Epidermal growth factor receptor erbB1	EGFR	P00533	CHEMBL203			Yes	Yes
Focal adhesion kinase 1	PTK2	Q05397	CHEMBL2695			Yes	
G protein-coupled receptor kinase 6	GRK6	P43250	CHEMBL6144		Yes		
Glycogen synthase kinase-3 alpha	GSK3A	P49840	CHEMBL2850			Yes	
Glycogen synthase kinase-3 beta	GSK3B	P49841	CHEMBL262			Yes	
Inhibitor of nuclear factor kappa B kinase epsilon subunit	IKBKE	Q14164	CHEMBL3529	Yes			
Kinesin-1 heavy chain/ Tyrosine-protein kinase receptor RET	RET	P07949	CHEMBL2041				Yes
LIM domain kinase 1	LIMK1	P53667	CHEMBL3836				Yes
LIM domain kinase 2	LIMK2	P53671	CHEMBL5932				Yes
MAP kinase ERK2	MAPK1	P28482	CHEMBL4040	Yes		Yes	

MAP kinase p38 beta	MAPK11	Q15759	CHEMBL3961				Yes
MAP kinase signal-integrating kinase 2	MKNK2	Q9HBH9	CHEMBL4204			Yes	
Maternal embryonic leucine zipper kinase	MELK	Q14680	CHEMBL4578				Yes
Mitogen-activated protein kinase kinase kinase kinase 4	MAP4K4	O95819	CHEMBL6166			Yes	
Platelet-derived growth factor receptor beta	PDGFRB	P09619	CHEMBL1913				Yes
Protein kinase C alpha	PRKCA	P17252	CHEMBL299		Yes		
Protein kinase C beta	PRKCB	P05771	CHEMBL3045		Yes		
Protein kinase C delta	PRKCD	Q05655	CHEMBL2996		Yes		
Protein kinase C epsilon	PRKCE	Q02156	CHEMBL3582		Yes		
Protein kinase C eta	PRKCH	P24723	CHEMBL3616		Yes		
Protein kinase C gamma	PRKCG	P05129	CHEMBL2938		Yes		
Proto-oncogene tyrosine-protein kinase MER	MERTK	Q12866	CHEMBL5331	Yes			
Pyruvate dehydrogenase kinase isoform 1	PDK1	Q15118	CHEMBL4766			Yes	Yes
Receptor protein-tyrosine kinase erbB-2	ERBB2	P04626	CHEMBL1824		Yes		Yes
Rho-associated protein kinase 1	ROCK1	Q13464	CHEMBL3231	Yes		Yes	Yes
Rho-associated protein kinase 2	ROCK2	O75116	CHEMBL2973	Yes	Yes		Yes
Ribosomal protein S6 kinase 1	RPS6KB1	P23443	CHEMBL4501			Yes	
Ribosomal protein S6 kinase alpha 1	RPS6KA1	Q15418	CHEMBL2553			Yes	
Serine/threonine-protein kinase 17B	STK17B	O94768	CHEMBL3980	Yes			Yes
Serine/threonine-protein kinase Aurora-A	AURKA	O14965	CHEMBL4722	Yes		Yes	Yes
Serine/threonine-protein kinase Aurora-B	AURKB	Q96GD4	CHEMBL2185	Yes		Yes	Yes
Serine/threonine-protein kinase B-raf	BRAF	P15056	CHEMBL5145	Yes		Yes	Yes
Serine/threonine-protein kinase Chk1	CHEK1	O14757	CHEMBL4630	Yes		Yes	Yes
Serine/threonine-protein kinase Chk2	CHEK2	O96017	CHEMBL2527	Yes	Yes		
Serine/threonine-protein kinase mTOR	MTOR	P42345	CHEMBL2842			Yes	
Serine/threonine-protein kinase PLK1	PLK1	P53350	CHEMBL3024	Yes			
Serine/threonine-protein kinase RAF	RAF1	P04049	CHEMBL1906			Yes	Yes
Serine/threonine-protein kinase Sgk1	SGK1	O00141	CHEMBL2343			Yes	
Serine/threonine-protein kinase TAO1	TAOK1	Q7L7X3	CHEMBL5261	Yes			
Serine/threonine-protein kinase TAO3	TAOK3	Q9H2K8	CHEMBL5701	Yes			
Serine/threonine-protein kinase TBK1	TBK1	Q9UHD2	CHEMBL5408	Yes			
Serine/threonine-protein kinase WEE1	WEE1	P30291	CHEMBL5491			Yes	
Serine/threonine-protein kinase/endoribonuclease IRE1	ERN1	O75460	CHEMBL1163101	Yes		Yes	Yes

Sphingosine kinase 1	SPHK1	Q9NYA1	CHEMBL4394			Yes	
Sphingosine kinase 2	SPHK2	Q9NRA0	CHEMBL3023			Yes	
Tyrosine-protein kinase ABL	ABL1	P00519	CHEMBL1862		Yes		Yes
Tyrosine-protein kinase ITK/TSK	ITK	Q08881	CHEMBL2959				Yes
Tyrosine-protein kinase LCK	LCK	P06239	CHEMBL258				Yes
Tyrosine-protein kinase receptor FLT3	FLT3	P36888	CHEMBL1974	Yes			Yes
Tyrosine-protein kinase SYK	SYK	P43405	CHEMBL2599	Yes	Yes		Yes
Tyrosine-protein kinase SYK	SYK	P43405	CHEMBL2599			Yes	
Vascular endothelial growth factor receptor 1	FLT1	P17948	CHEMBL1868				Yes

A

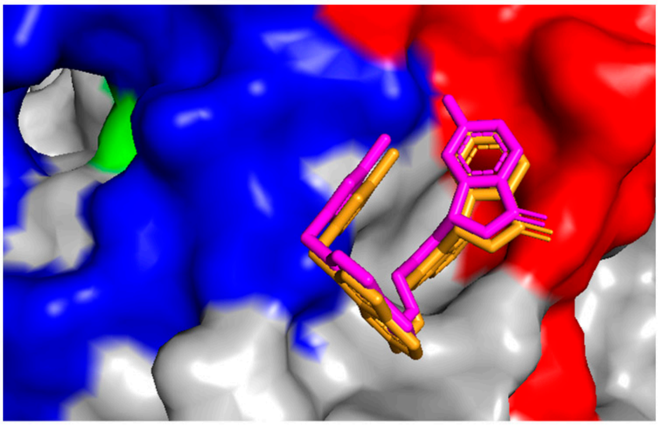

B

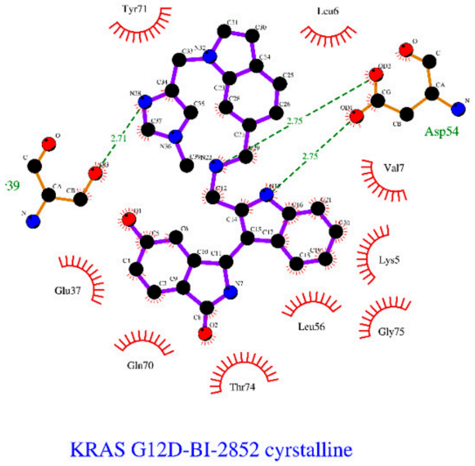

C

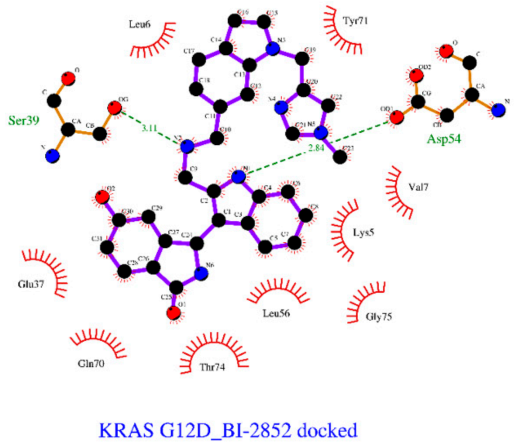

Supplementary Figure S1: Binding poses and molecular interactions of BI-2852 in crystallized and docked forms. [A] The P-loop (residues 10-17), Switch I (residues 25-40), and Switch II (residues 57-76) were shown in green, blue, and red color respectively, and the crystallized and docked forms of BI-2852 were shown in magenta and bright yellow color respectively. [B] Molecular interactions of BI-2852 in crystallized form. [C] Molecular interactions of BI-2852 in docked form. In both crystallized and docked forms, the Indole group near to the Isatin group of BI-2852 have closer interaction in the SI/II region, and also showed similar type of interactions with the residues of KRAS G12D mutant protein.

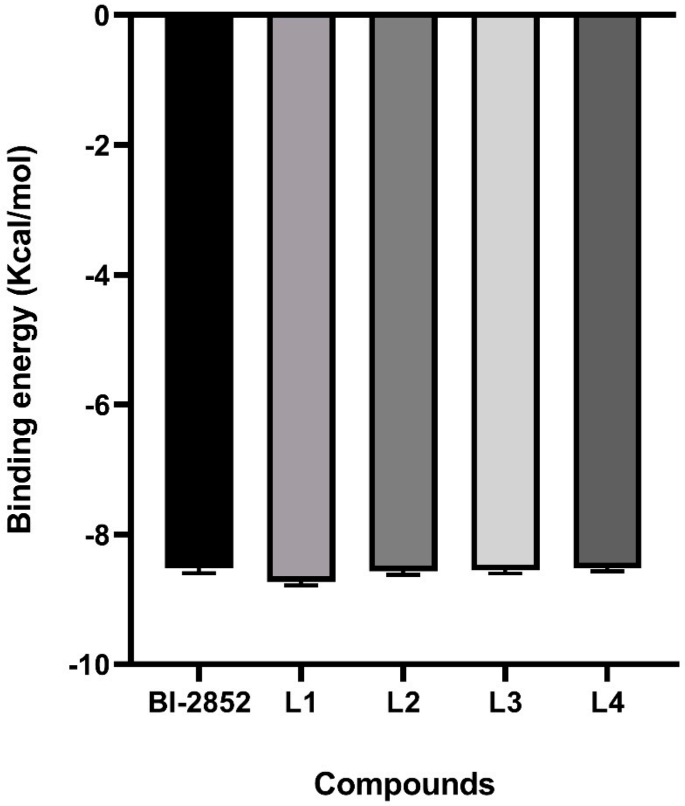

Supplementary Figure S2: Binding energies of BI-2852 and top 4 lead flavonoids against KRAS G12D mutant protein.
